# From reads to regions: a Bioconductor workflow to detect differential binding in ChIP-seq data

**DOI:** 10.12688/f1000research.7016.2

**Published:** 2016-01-11

**Authors:** Aaron T. L. Lun, Gordon K. Smyth

**Affiliations:** 1 The Walter and Eliza Hall Institute of Medical Research, Melbourne, Australia; 2 Department of Medical Biology, The University of Melbourne, Melbourne, Australia; 3 Department of Mathematics and Statistics, The University of Melbourne, Melbourne, Australia

**Keywords:** ChIP-seq, Differential binding, genomics, bioinformatics, read alignment, visualization

## Abstract

Chromatin immunoprecipitation with massively parallel sequencing (ChIP-seq) is widely used to identify the genomic binding sites for protein of interest. Most conventional approaches to ChIP-seq data analysis involve the detection of the absolute presence (or absence) of a binding site. However, an alternative strategy is to identify changes in the binding intensity between two biological conditions, i.e., differential binding (DB). This may yield more relevant results than conventional analyses, as changes in binding can be associated with the biological difference being investigated. The aim of this article is to facilitate the implementation of DB analyses, by comprehensively describing a computational workflow for the detection of DB regions from ChIP-seq data. The workflow is based primarily on R software packages from the open-source Bioconductor project and covers all steps of the analysis pipeline, from alignment of read sequences to interpretation and visualization of putative DB regions. In particular, detection of DB regions will be conducted using the counts for sliding windows from the csaw package, with statistical modelling performed using methods in the edgeR package. Analyses will be demonstrated on real histone mark and transcription factor data sets. This will provide readers with practical usage examples that can be applied in their own studies.

## Introduction

Chromatin immunoprecipitation with sequencing (ChIP-seq) is a popular technique for identifying the genomic binding sites of a target protein. Conventional analyses of ChIP-seq data aim to detect absolute binding (i.e., the presence or absence of a binding site) based on peaks in the read coverage. However, a number of recent studies have focused on the detection of changes in the binding profile between conditions ( Pal *et al.*, 2013; Ross-Innes *et al.*, 2012). These differential binding (DB) analyses involve counting reads into genomic intervals, and then testing those counts for significant differences between conditions. This defines a set of putative DB regions for further examination. DB analyses are easier to perform than their conventional counterparts, as the effect of genomic biases is largely mitigated when counts for different libraries are compared at the same genomic region. DB regions may also be more relevant as the change in binding can be associated with the biological difference between conditions.

The key step in the DB analysis is the manner in which reads are counted. The most obvious strategy is to count reads into pre-defined regions of interest, like promoters or gene bodies ( Pal *et al.*, 2013). This is simple but will not capture changes outside of those regions. In contrast, *de novo* analyses do not depend on pre-specified regions, instead using empirically defined peaks or sliding windows for read counting. Peak-based methods are implemented in the *DiffBind* and *DBChIP* software packages ( Liang & Keles, 2012; Ross-Innes *et al.*, 2012), which count reads into peak intervals that have been identified with software like MACS ( Zhang *et al.*, 2008). This requires some care to maintain statistical rigour, as peaks are called with the same data used to test for DB. Alternatively, window-based approaches count reads into sliding windows across the genome. This is a more direct strategy that avoids problems with data re-use and can provide increased DB detection power ( Lun & Smyth, 2014). However, its correct implementation is not straightforward due to the subtleties with interpretation of the false discovery rate (FDR).

This article describes a computational workflow for performing a DB analysis with sliding windows. The aim is to facilitate the practical implementation of window-based DB analyses by providing detailed code and expected output. The workflow described here applies to any ChIP-seq experiment with multiple experimental conditions and with multiple biological samples within one or more of the conditions. It detects and summarizes DB regions between conditions in a *de novo* manner, i.e., without making any prior assumptions about the location or width of bound regions. Detected regions are then annotated according to their proximity to annotated genes. In addition, the code can be easily adapted to accommodate batch effects, covariates and multiple experimental factors.

The workflow is based primarily on software packages from the open-source Bioconductor project ( Huber *et al.*, 2015). It contains all steps that are necessary for detecting DB regions, starting from the raw read sequences. Reads are first aligned to the genome using the *Rsubread* package ( Liao *et al.*, 2013). These are counted into sliding windows with *csaw*, to quantify binding intensity across the genome ( Lun & Smyth, 2014; Lun & Smyth, 2015). Statistical modelling is based on the negative binomial (NB) distribution with generalized linear models (GLMs) in the *edgeR* package ( McCarthy *et al.*, 2012; Robinson *et al.*, 2010), with additional sophistication provided by quasi-likelihood (QL) methods ( Lund *et al.*, 2012). Code is also provided for filtering, normalization and region-level control of the FDR. Finally, annotation and visualization of the DB regions is described using *Gviz* and other packages.

The application of the methods in this article will be demonstrated on two publicly available ChIP-seq data sets. The first data set studies changes in H3K9ac marking between pro-B and mature B cells ( Revilla-I-Domingo *et al.*, 2012). The second data set studies changes in CREB-binding protein (CBP) binding between wild-type and CBP knock-out cells ( Kasper *et al.*, 2014). These two studies were chosen to represent common situations where a DB analysis can be applied – one involving sharp binding with CBP, and the other involving broader marking with H3K9ac. A separate workflow is described for the analysis of each data set, using the sliding window approach in both cases but with different parameter settings. The intention is to provide readers with a variety of usage examples from which they can construct DB analyses of their own data.

## Aligning reads in the H3K9ac libraries

The first task is to download the relevant ChIP-seq libraries from the NCBI Gene Expression Omnibus (GEO) ( Edgar *et al.*, 2002). These are obtained from the data series GSE38046, using the Sequence Read Accession (SRA) numbers listed below. The experiment contains two biological replicates in total for each of the two cell types, i.e., pro-B and mature B. Multiple technical replicates exist for some of the biological replicates, and are indicated as those files with the same grouping.



                    sra.numbers <- 
                    c
                    (
                    "SRR499718"
                    , 
                    "SRR499719"
                    , 
                    "SRR499720"
                    , 
                    "SRR499721"
                    ,
    
                    "SRR499734"
                    , 
                    "SRR499735"
                    , 
                    "SRR499736"
                    , 
                    "SRR499737"
                    , 
                    "SRR499738"
                    )

                    grouping <- 
                    c
                    (
                    "proB-8113"
                    , 
                    "proB-8113"
                    , 
                    "proB-8108"
                    , 
                    "proB-8108"
                    ,
    
                    "matureB-8059"
                    , 
                    "matureB-8059"
                    , 
                    "matureB-8059"
                    , 
                    "matureB-8059"
                    , 
                    "matureB-8086"
                    )
all.sra <- 
                    paste0
                    (sra.numbers, 
                    ".lite.sra"
                    )

                    data.frame
                    (
                    SRA=
                    all.sra, 
                    Condition=
                    grouping)
                




                    ##		    SRA	   Condition
## 1 SRR499718.lite.sra	   proB-8113
## 2 SRR499719.lite.sra	   proB-8113
## 3 SRR499720.lite.sra	   proB-8108
## 4 SRR499721.lite.sra	   proB-8108
## 5 SRR499734.lite.sra matureB-8059
## 6 SRR499735.lite.sra matureB-8059
## 7 SRR499736.lite.sra matureB-8059
## 8 SRR499737.lite.sra matureB-8059
## 9 SRR499738.lite.sra matureB-8086
                


These files are downloaded in the SRA format, and need to be unpacked to the FASTQ format prior to alignment. This can be done using the fastq-dump utility from the SRA Toolkit.



                    for (sra in all.sra) {
    code <- 
                    system
                    (
                    paste
                    (
                    "fastq-dump"
                    , sra))
     
                    stopifnot
                    (code==0L)
}
all.fastq <- 
                    paste0
                    (sra.numbers, 
                    ".fastq"
                    )
                


Reads from technical replicates are pooled together into a single FASTQ file prior to further processing. This reflects the fact that they originate from a single library of DNA fragments.



                    by.group <- 
                    split
                    (all.fastq, grouping)
for (group in 
                    names
                    (by.group)) {
    code <- 
                    system
                    (
                    paste
                    (
                    c
                    (
                    "cat"
                    , by.group[[group]], 
                    ">"
                    ,
          
                    paste0
                    (group, 
                    ".fastq"
                    )), 
                    collapse=
                    " "
                    ))
     
                    stopifnot
                    (code==0L)
}
group.fastq <- 
                    paste0
                    (
                    names
                    (by.group), 
                    ".fastq"
                    )
                


Reads in each library are aligned to the mm10 build of the mouse genome, using the align function in the *Rsubread* package ( Liao *et al.*, 2013). This assumes that an index has already been constructed with the prefix index/mm10. The function uses a seed-and-vote paradigm to quickly and accurately map reads to the genome by focusing on locations that receive a number of votes above some consensus threshold. Here, a threshold of 2 votes is used instead of the default of 3, to accommodate the short length of the reads (32–36 bp). The type parameter is also set to optimize for genomic alignment, rather than alignment to the transcriptome.



                    library
                    (Rsubread)
bam.files <- 
                    paste0
                    (
                    names
                    (by.group), 
                    ".bam"
                    )

                    align
                    (
                    index=
                    "index/mm10"
                    , 
                    readfile1=
                    group.fastq, 
                    TH1=
                    2
                    , 
                    type=
                    1
                    ,
    
                    input_format=
                    "FASTQ"
                    , 
                    output_file=
                    bam.files)
                


In each of the resulting BAM files, alignments are re-sorted by their mapping locations. This is required for input into *csaw*, but is also useful for other programs like genome browsers that depend on sorting and indexing for rapid retrieval of reads.



                    library
                    (Rsamtools)
for (bam in bam.files) {
    out <- 
                    suppressWarnings
                    (
                    sortBam
                    (bam, 
                    "h3k9ac_temp"
                    ))
    
                    file.rename
                    (out, bam)
}
                


Potential PCR duplicates are marked using the MarkDuplicates tool from the Picard software suite. These are identified as alignments at the same genomic location, such that they may have originated from PCR-amplified copies of the same DNA fragment.



                    temp.bam <- 
                    "h3k9ac_temp.bam"

                    temp.file <- 
                    "h3k9ac_metric.txt"

                    temp.dir <- 
                    "h3k9ac_working"

                    dir.create
                    (temp.dir)
for (bam in bam.files) {
    code <- 
                    system
                    (
                    sprintf
                    (
       
                    "MarkDuplicates I=%s O=%s M=%s \\
      TMP_DIR=%s AS=true REMOVE_DUPLICATES=false \\
      VALIDATION_STRINGENCY=SILENT"
                    , bam, temp.bam,
      temp.file, temp.dir))
     
                    stopifnot
                    (code==0L)
     
                    file.rename
                    (temp.bam, bam)
}
                


The behaviour of the alignment pipeline for this data set can be easily summarized with some statistics. Ideally, the proportion of mapped reads should be high (70–80% or higher), while the proportion of marked reads should be low (below 20%). Note that only reads with unique mapping locations are reported by *Rsubread* as being successfully mapped.



                    diagnostics <- 
                    list
                    ()

                    for (bam in bam.files) {
    total <- 
                    countBam
                    (bam)$records
    mapped <- 
                    countBam
                    (bam, 
                    param=ScanBamParam
                    (
	 
                    flag=scanBamFlag
                    (
                    isUnmapped=
                    FALSE
                    )))$records
    marked <- 
                    countBam
                    (bam, 
                    param=ScanBamParam
                    (
	 
                    flag=scanBamFlag
                    (
                    isUnmapped=
                    FALSE
                    , 
                    isDuplicate=
                    TRUE
                    )))$records
    diagnostics[[bam]] <- 
                    c
                    (
                    Total=
                    total, 
                    Mapped=
                    mapped, 
                    Marked=
                    marked)
}
diag.stats <- 
                    data.frame
                    (
                    do.call
                    (rbind, diagnostics))
diag.stats$Prop.mapped <- diag.stats$Mapped/diag.stats$Total*
                    100

                    diag.stats$Prop.marked <- diag.stats$Marked/diag.stats$Mapped*
                    100

                    diag.stats
                




                    ##		       Total  Mapped  Marked Prop.mapped Prop.marked
## matureB-8059.bam 16675372 7752077 1054591    46.48818   13.603980
## matureB-8086.bam  6347683 4899961  195100    77.19291    3.981664
## proB-8108.bam    10413135 8213980  297796	78.88095    3.625478
## proB-8113.bam    10724526 9145743  489177	85.27876    5.348685
                


Finally, the libraries are indexed for rapid retrieval by genomic location. This generates a number of index files at the same location as the BAM files.



                    indexBam
                    (bam.files)
                


## Obtaining the ENCODE blacklist for mm10

A number of genomic regions contain high artifactual signal in ChIP-seq experiments. These often correspond to genomic features like telomeres or microsatellite repeats. For example, multiple tandem repeats in the real genome are reported as a single unit in the genome build. Alignment of all (non-specifically immunoprecipitated) reads from the former will result in artificially high coverage of the latter. Moreover, differences in repeat copy numbers between conditions can lead to detection of spurious DB.

As such, these regions must be removed prior to further analysis. This can be done with an annotated blacklist of problematic regions in the mm9 build of the mouse genome. All reads in the blacklist will be ignored during processing in *csaw*. The blacklist itself was constructed by identifying consistently problematic regions in the ENCODE and modENCODE data sets ( ENCODE Project Consortium, 2012).

Recall that the alignments have been performed to the mm10 build, so the mm9 blacklist coordinates must be transferred to their mm10 equivalents. This is done using the liftOver function in the *rtracklayer* package ( Lawrence *et al.*, 2009). The chain file specifies the corresponding coordinates between the two builds and can be obtained here. The new blacklist coordinates are then saved to file for future use.



                    library
                    (rtracklayer)
ch <- 
                    import.chain
                    (
                    "mm9ToMm10.over.chain"
                    )
original <- 
                    import
                    (
                    "mm9-blacklist.bed"
                    )
blacklist <- 
                    liftOver
                    (
                    x=
                    original, 
                    chain=
                    ch)
blacklist <- 
                    unlist
                    (blacklist)

                    saveRDS
                    (
                    file=
                    "mm10-blacklist.rds"
                    , blacklist)
                


Any user-defined set of regions can be used as a blacklist in this analysis. For example, one could use predicted repeat regions from the UCSC genome annotation ( Rosenbloom *et al.*, 2015). This tends to remove a greater number of problematic regions (especially microsatellites) compared to the ENCODE blacklist. However, the size of the UCSC list means that genuine DB sites may also be removed. Thus, the ENCODE blacklist is preferred for most applications. Alternatively, if negative control libraries are available, they can be used to empirically identify problematic regions with the *GreyListChIP* package. These regions should be ignored as they have high coverage in the controls and are unlikely to be genuine binding sites.

## Testing for DB between pro-B and mature B cells

### Setting up the analysis parameters

Here, the settings for the DB analysis are specified. Recall that the paths to the BAM files are stored in the bam.files vector after alignment. The cell type for each file can be conveniently extracted from the file name.



                        celltype <- 
                        sub
                        (
                        "-.*"
                        ,  
                        ""
                        , bam.files)

                        data.frame
                        (
                        BAM=
                        bam.files,  
                        CellType=
                        celltype)
                    




                        ##                BAM  CellType
## 1 matureB-8059.bam   matureB
## 2 matureB-8086.bam   matureB
## 3    proB-8108.bam      proB
## 4    proB-8113.bam      proB
                    


In the *csaw* package, the readParam object determines which reads are extracted from the BAM files. The idea is to set this up once and to re-use it in all relevant functions. For this analysis, reads are only used if they have a mapping quality (MAPQ) score equal to or above 50. This avoids spurious results due to weak or non-unique alignments. While a MAPQ threshold of 50 is quite conservative, a stringent threshold is necessary here due to the short length of the reads. Reads are also ignored if they map within blacklist regions or if they do not map to the standard set of mouse nuclear chromosomes.



                        library
                        (csaw)

                        standard.chr <- 
                        paste0
                        (
                        "chr"
                        , 
                        c
                        (
                        1
                        :
                        19
                        , 
                        "X"
                        , 
                        "Y"
                        ))

                        param <- 
                        readParam
                        (
                        minq=
                        50
                        , 
                        discard=
                        blacklist,
                        restrict=standard.chr)


### Computing the average fragment length

**Figure 1.**
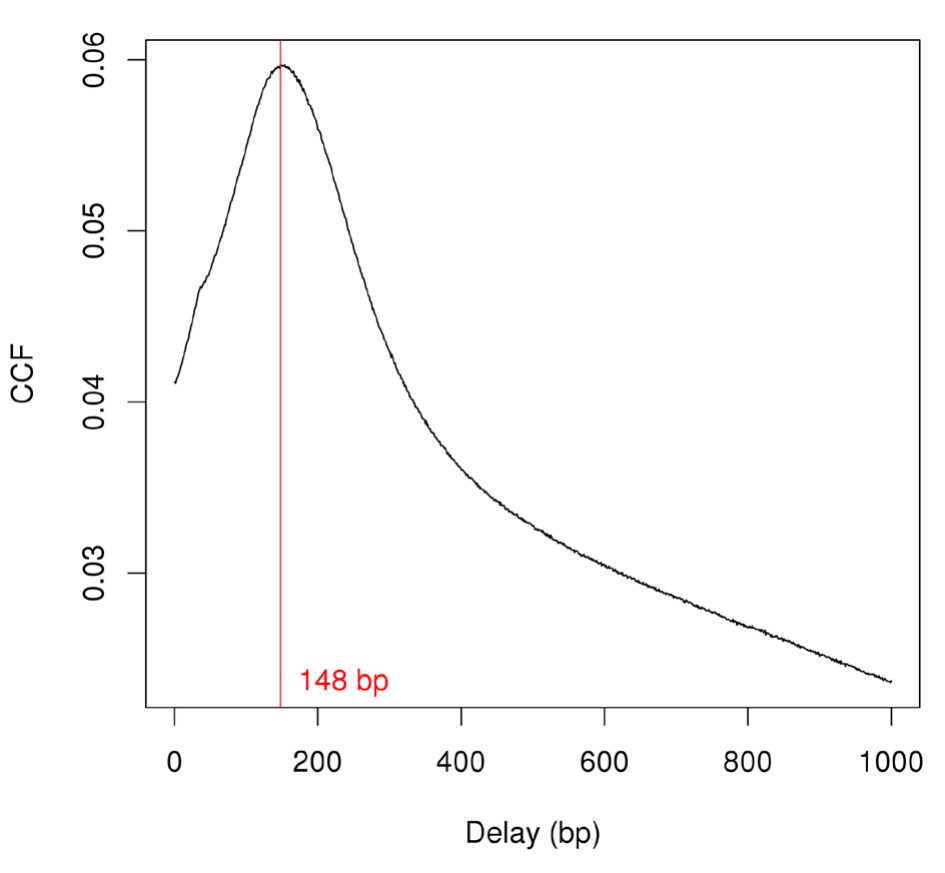
Cross-correlation function (CCF) against delay distance for the H3k9ac data set. The delay with the maximum correlation is shown as the red line.

Strand bimodality is often observed in ChIP-seq experiments involving narrow binding events like H3K9ac marking. This refers to the presence of distinct subpeaks on each strand and can be quantified with cross-correlation plots ( Kharchenko *et al.*, 2008). A strong peak in the cross-correlations should be observed if immunoprecipitation was successful. The delay distance at the peak corresponds to the distance between forward-/reverse-strand subpeaks. This is identified from Figure 1 and is used as the average fragment length for this analysis.



                        x <- 
                        correlateReads
                        (bam.files, 
                        param=reform
                        (param, 
                        dedup=
                        TRUE
                        ))
frag.len <- 
                        which.max
                        (x) - 
                        1

                        frag.len
                    




                        ## [1] 148
                    




                        plot
                        (
                        1:
                        length
                        (x)-
                        1
                        , x, 
                        xlab=
                        "Delay (bp)"
                        , 
                        ylab=
                        "CCF"
                        , 
                        type=
                        "l"
                        )

                        abline(
                        v=
                        frag.len, 
                        col=
                        "red"
                        )

                        text
                        (
                        x=
                        frag.len, 
                        y=min
                        (x), 
                        paste
                        (frag.len, 
                        "bp"
                        ), 
                        pos=
                        4
                        , 
                        col=
                        "red"
                        )
                    


Only unmarked reads (i.e., not potential PCR duplicates) are used here. This tends to give better signal by reducing the size of the “phantom” peak at the read length ( Landt *et al.*, 2012). However, removal of marked reads is risky as it caps the signal in high-coverage regions of the genome. This can result in loss of power to detect DB, or introduction of spurious DB when the same cap is applied to libraries of different sizes. Thus, the marking status of each read will be ignored in the rest of the analysis, i.e., no duplicates will be removed in downstream steps.

### Counting reads into windows

*csaw* uses a sliding window strategy to quantify binding intensity across the genome. Each read is directionally extended to the average fragment length, to represent the DNA fragment from which that read was sequenced. The number of extended reads overlapping a window is counted. The window is then moved to its next position on the genome, and counting is repeated. (Each read is usually counted into multiple windows, which will introduce correlations between adjacent windows but will not otherwise affect the analysis.) This is done for all libraries such that a count is obtained for each window in each library. The windowCounts function produces a RangedSummarizedExperiment object containing these counts in matrix form, where each row corresponds to a window and each column represents a library.



                        win.data <- 
                        windowCounts
                        (bam.files, 
                        param=
                        param, 
                        width=
                        150
                        , 
                        ext=
                        frag.len)
win.data
                    




                        ## class: RangedSummarizedExperiment
## dim: 1569624 4
## metadata(4): spacing width shift final.ext
## assays(1): counts
## rownames: NULL
## rowRanges metadata column names(0):
## colnames: NULL
## colData names(4): bam.files totals ext param
                    


To analyze H3K9ac data, a window size of 150 bp is used here. This corresponds roughly to the length of the DNA in a nucleosome ( Humburg *et al.*, 2011), which is the smallest relevant unit for studying histone mark enrichment. The spacing between windows is set to the default of 50 bp, i.e., the start positions for adjacent windows are 50 bp apart. Smaller spacings can be used to improve spatial resolution, but will increase memory usage and runtime by increasing the number of windows required to cover the genome. This is unnecessary as increased resolution confers little practical benefit for this data set – counts for very closely spaced windows will be practically identical. Finally, windows with very low counts (by default, less than a sum of 10 across all libraries) are removed to reduce memory usage. This represents a preliminary filter to remove uninteresting windows corresponding to likely background regions.

### Filtering windows by abundance

As previously mentioned, low-abundance windows contain no binding sites and need to be filtered out. This improves power by removing irrelevant tests prior to the multiple testing correction; avoids problems with discreteness in downstream statistical methods; and reduces computational work for further analyses. Here, filtering is performed using the average abundance of each window ( McCarthy *et al.*, 2012), which is defined as the average log-count per million for that window. This performs well as an independent filter statistic for NB-distributed count data ( Lun & Smyth, 2014).

The filter threshold is defined based on the assumption that most regions in the genome are not marked by H3K9ac. Reads are counted into large bins and the median coverage across those bins is used as an estimate of the background abundance. This estimate is then compared to the average abundances of the windows, after rescaling to account for differences in the window and bin sizes. A window is only retained if its coverage is 3-fold higher than that of the background regions, i.e., the abundance of the window is greater than the background abundance estimate by log _2_(3) or more. This removes a large number of windows that are weakly or not marked and are likely to be irrelevant.



                        bins <- 
                        windowCounts
                        (bam.files, 
                        bin=
                        TRUE
                        , 
                        width=
                        2000
                        , 
                        param=
                        param)
filter.stat <- 
                        filterWindows
                        (win.data,  bins, 
                        type=
                        "global"
                        )
min.fc <- 
                        3

                        keep <- filter.stat$filter > 
                        log2
                        (min.fc)

                        summary
                        (keep)
                    




                        ##     Mode      FALSE    TRUE   NA's
##  logical	906406	663218	    0
                    


**Figure 2.**
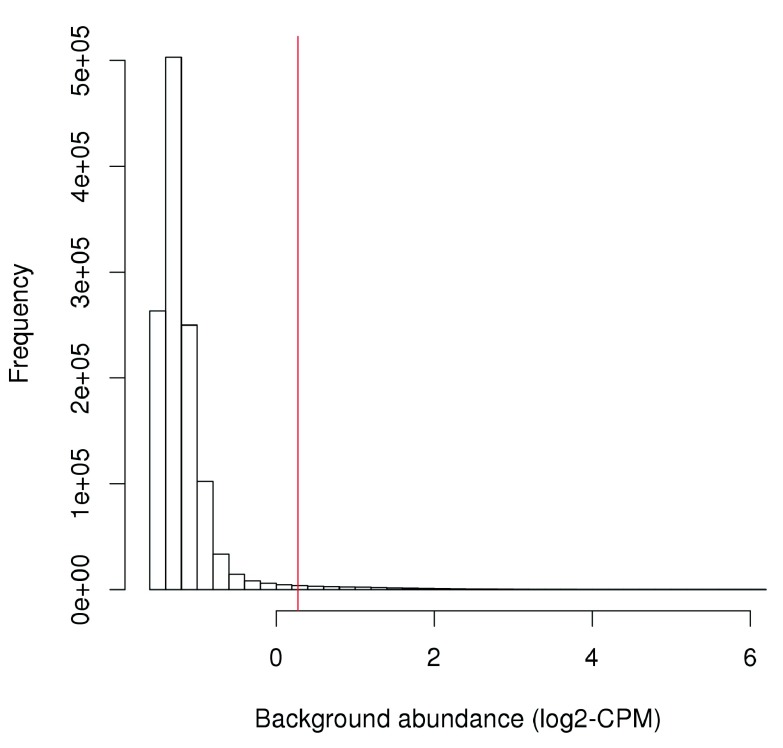
Histogram of average abundances across all 2 kbp genomic bins. The filter threshold is shown as the red line.



The effect of the fold-change threshold can be examined visually in Figure 2. The chosen threshold is greater than the abundances of most bins in the genome – presumably, those that contain background regions. This suggests that the filter will remove most windows lying within background regions.

                        hist
                        (filter.stat$back.abundances, 
                        main=
                        ""
                        , 
                        breaks=
                        50
                        ,
     
                        xlab=
                        "Background abundance (log2-CPM)"
                        )
threshold <- filter.stat$abundances[
                        1
                        ] - filter.stat$filter[
                        1
                        ] + 
                        log2
                        (min.fc)

                        abline
                        (
                        v=
                        threshold, 
                        col=
                        "red"
                        )
                    


The actual filtering itself is done by simply subsetting the RangedSummarizedExperiment object.



                        filtered.data <- win.data[keep,]
                    


### Normalizing for library-specific trended biases

**Figure 3.**
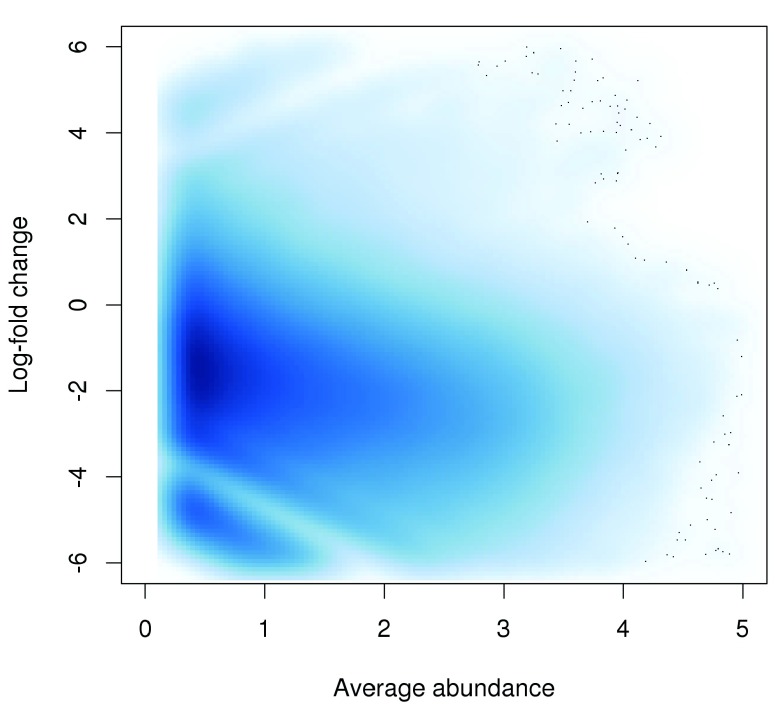
Abundance-dependent trend in the log-fold change between two H3K9ac libraries (mature B over pro-B), across all windows retained after filtering.



Normalization is required to eliminate confounding library-specific biases prior to any comparisons between libraries. In particular, a trended bias is often observed between libraries in Figure 3. This refers to a systematic fold-difference in window coverage between libraries that changes according to the average abundance of the window.

                        win.ab <- filter.stat$abundances[keep]
adjc <- 
                        log2
                        (
                        assay
                        (filtered.data)+
                        0.5
                        )
logfc <- adjc[,
                        1
                        ] - adjc[,
                        4
                        ]

                        smoothScatter
                        (win.ab, logfc, 
                        ylim=c
                        (-
                        6
                        , 
                        6
                        ), 
                        xlim=c
                        (
                        0
                        , 
                        5
                        ),
     
                        xlab=
                        "Average abundance"
                        , 
                        ylab=
                        "Log-fold change"
                        )
                    


Trended biases cannot be removed by scaling methods like TMM normalization ( Robinson & Oshlack, 2010), as the amount of scaling required varies with the abundance of the window. Rather, non-linear normalization methods must be used. *csaw* implements a version of the fast loess method ( Ballman *et al.*, 2004) that has been modified to handle count data ( Lun & Smyth, 2015). This produces a matrix of offsets that can be used during GLM fitting.



                        offsets <- 
                        normOffsets
                        (filtered.data, 
                        type=
                        "loess"
                        )

                        head
                        (offsets)
                    




                        ##	      [,1]       [,2]      [,3]      [,4]
## [1,] -0.5878496 -0.4019382 0.3954267 0.5943611
## [2,] -0.5673338 -0.3789731 0.3770978 0.5692091
## [3,] -0.6261679 -0.4720746 0.4397909 0.6584516
## [4,] -0.6528790 -0.5453416 0.4789700 0.7192507
## [5,] -0.6713098 -0.5838111 0.5015881 0.7535328
## [6,] -0.7028331 -0.6463783 0.5390876 0.8101237
                    


**Figure 4.**
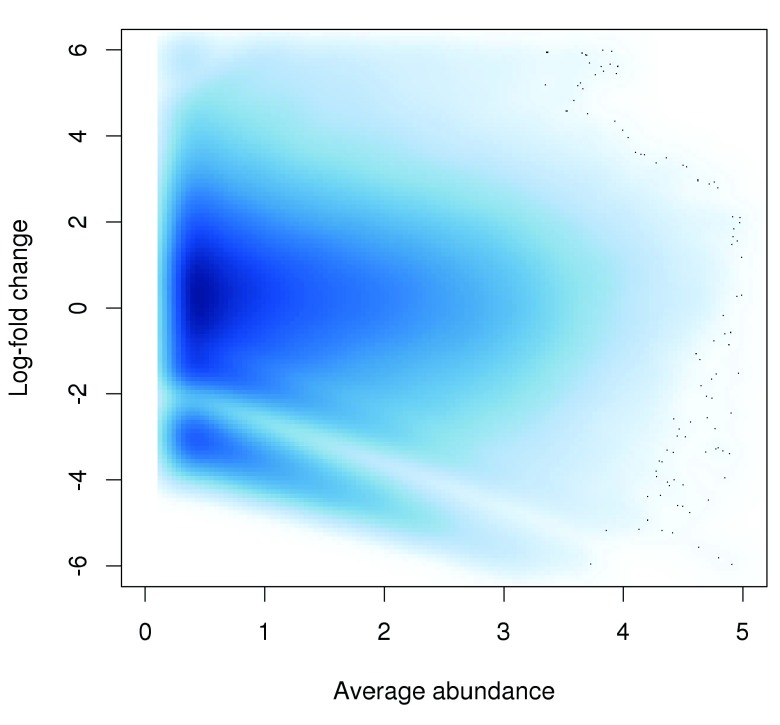
Effect of non-linear normalization on the trended bias between two H3K9ac libraries. Normalized log-fold changes are shown for all windows retained after filtering.

The effect of non-linear normalization can be visualized with a mean-difference plot comparing the first and last libraries. Once the offsets are applied to adjust the log-fold changes, the trend is eliminated from the plot ( Figure 4). The cloud of points is also centred at a log-fold change of zero. This indicates that normalization was successful in removing the differences between libraries.



                        norm.adjc <- adjc - offsets/
                        log
                        (
                        2
                        )

                        norm.fc <- norm.adjc[,
                        1
                        ]-norm.adjc[,
                        4
                        ]

                        smoothScatter
                        (win.ab, norm.fc, 
                        ylim=c
                        (-
                        6
                        , 
                        6
                        ), 
                        xlim=c
                        (
                        0
                        , 
                        5
                        ),
     
                        xlab=
                        "Average abundance"
                        , 
                        ylab=
                        "Log-fold change"
                        )
                    


The implicit assumption of non-linear methods is that most windows at each abundance are not DB. Any systematic difference between libraries is attributed to bias and is removed. The assumption of a non-DB majority is reasonable for this data set, given that the cell types being compared are quite closely related. However, it is not appropriate in situations where large-scale DB is expected, as removal of the difference would result in loss of genuine DB. An alternative normalization strategy for these situations will be described later in the CBP analysis.

### Statistical modelling of biological variability

***Introduction.*** Counts are modelled using NB GLMs in the *edgeR* package ( McCarthy *et al.*, 2012; Robinson *et al.*, 2010). The NB distribution is useful as it can handle low, discrete counts for each window. The NB dispersion parameter allows modelling of biological variability between replicate libraries. GLMs can also accommodate complex experimental designs, though a simple design is sufficient for this study.



                        celltype <- 
                        factor
                        (celltype)
design <- 
                        model.matrix
                        (~
                        0
                        +celltype)

                        colnames
                        (design) <- 
                        levels
                        (celltype)

                        design
                    




                        ##    matureB proB
##  1	    1	 0
##  2	    1	 0
##  3	    0	 1
##  4	    0	 1
##  attr(,"assign")
##  [1] 1 1
##  attr(,"contrasts")
##  attr(,"contrasts")$celltype
##  [1] "contr.treatment"
                    


As a general rule, the experimental design should contain at least two replicates in each of the biological conditions. This ensures that the results for each condition are replicable and are not the result of technical artifacts such as PCR duplicates. Obviously, more replicates will provide more power to detect DB accurately and reliability, albeit at the cost of time and experimental resources.

***Estimating the NB dispersion.*** The RangedSummarizedExperiment object is coerced into a DGEList object (plus offsets) prior to entry into *edgeR*. Estimation of the NB dispersion is then performed. Specifically, a NB dispersion trend is fitted to all windows against the average abundance. This means that empirical mean-dispersion trends can be flexibly modelled.



                        library
                        (edgeR)
y <- 
                        asDGEList
                        (filtered.data)
y$offset <- offsets
y <- 
                        estimateDisp
                        (y, design)

                        summary
                        (y$trended.dispersion)
                    




                        ##     Min.  1st Qu.   Median     Mean  3rd Qu.     Max.
##  0.03156  0.04174  0.04274  0.04168  0.04311  0.04371
                    


**Figure 5.**
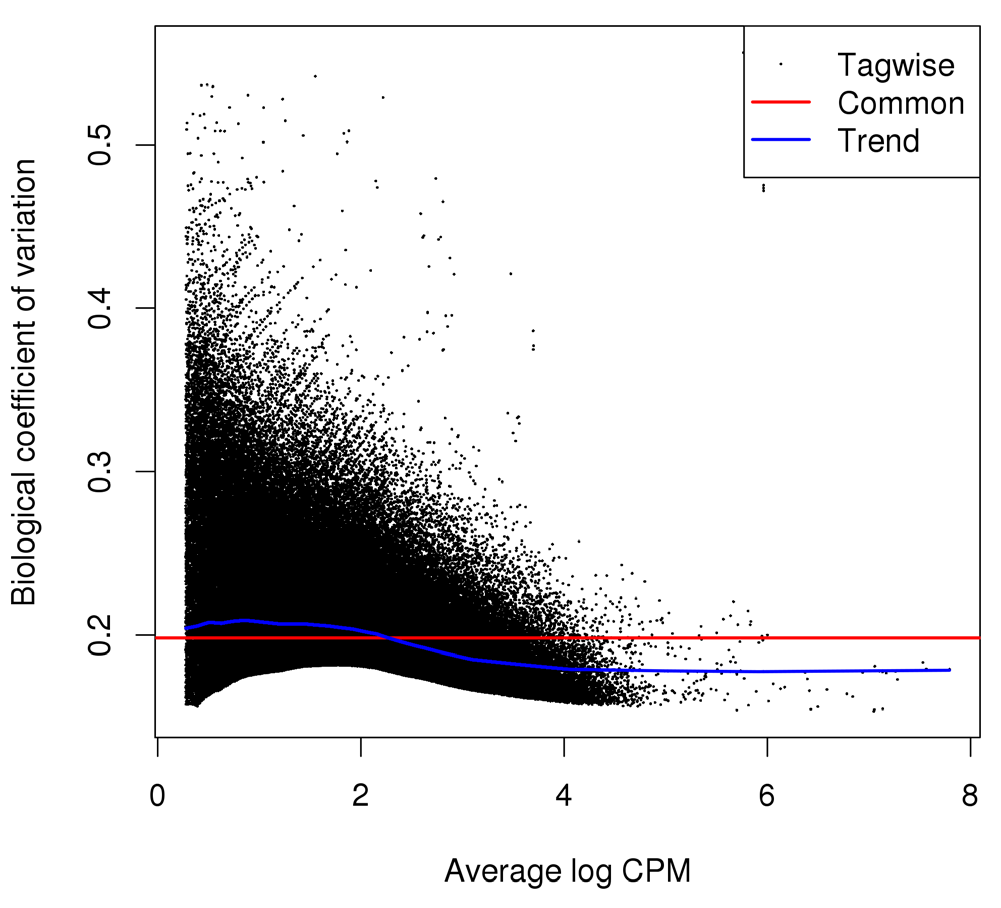
Abundance-dependent trend in the BCV for each window, represented by the blue line. Common (red) and tagwise estimates (black) are also shown.



The NB dispersion trend is visualized in Figure 5 as the biological coefficient of variation (BCV), i.e., the square root of the NB dispersion. Note that only the trended dispersion will be used in the downstream steps – the common and tagwise values are only shown for diagnostic purposes. Specifically, the common BCV provides an overall measure of the variability in the data set, averaged across all windows. Data sets with common BCVs ranging from 10 to 20% are considered to have low variability, i.e., counts are highly reproducible. The tagwise BCVs should also be dispersed above and below the fitted trend, indicating that the fit was successful.

                        plotBCV
                        (y)
                    


For most data sets, one would expect to see a trend that decreases to a plateau with increasing average abundance. This reflects the greater reliability of large counts, where the effects of stochasticity and technical artifacts (e.g., mapping errors, PCR duplicates) are averaged out. In Figure 5, the range of abundances after filtering is such that the plateau has already been reached. This is still a satisfactory result, as it indicates that the retained windows have low variability and more power to detect DB.

**Figure 6.**
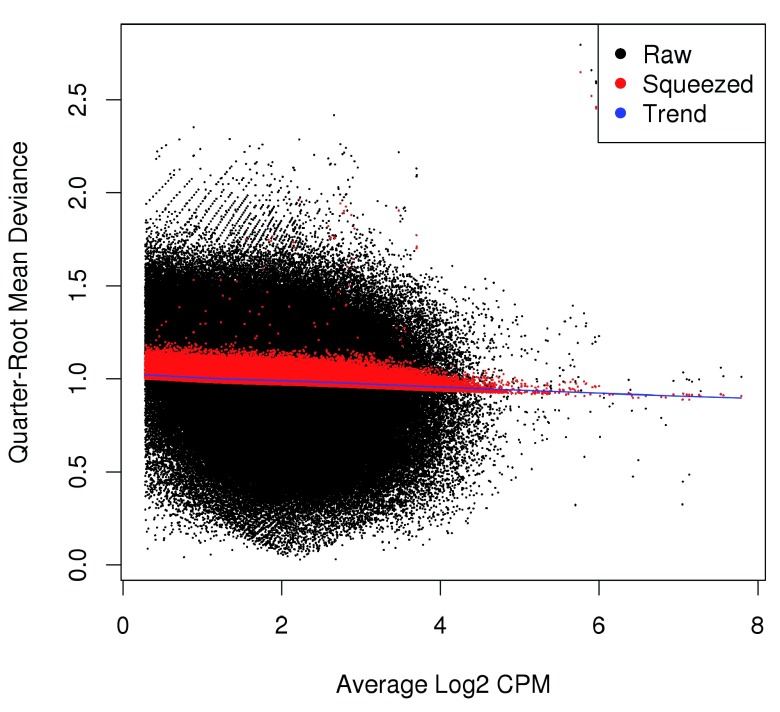
Effect of EB shrinkage on the raw QL dispersion estimate for each window (black) towards the abundance-dependent trend (blue) to obtain squeezed estimates (red).



***Estimating the QL dispersion.*** Additional modelling is provided with the QL methods in *edgeR* ( Lund *et al.*, 2012). This introduces a QL dispersion parameter for each window, which captures variability in the NB dispersion around the fitted trend for each window. Thus, the QL dispersion can model window-specific variability, whereas the NB dispersion trend is averaged across many windows. However, with limited replicates, there is not enough information for each window to stably estimate the QL dispersion. This is overcome by sharing information between windows with empirical Bayes (EB) shrinkage. The instability of the QL dispersion estimates is reduced by squeezing the estimates towards an abundance-dependent trend ( Figure 6).

                        fit <- 
                        glmQLFit
                        (y, design, 
                        robust=
                        TRUE
                        )

                        plotQLDisp
                        (fit)
                    


The extent of shrinkage is determined by the prior degrees of freedom (d.f.). Large prior d.f. indicates that the dispersions were similar across windows, such that strong shrinkage to the trend could be performed to increase stability and power. Small prior d.f. indicates that the dispersions were more variable. In such cases, less squeezing is performed as strong shrinkage would be inappropriate. Also note the use of robust=TRUE, which reduces the sensitivity of the EB procedures to outlier windows.



                        summary
                        (fit$df.prior)
                    




                        ##    Min.  1st Qu.   Median     Mean  3rd Qu.     Max.
##  0.4903  22.6900  22.6900  22.6900  22.6900  22.6900
                    


**Figure 7.**
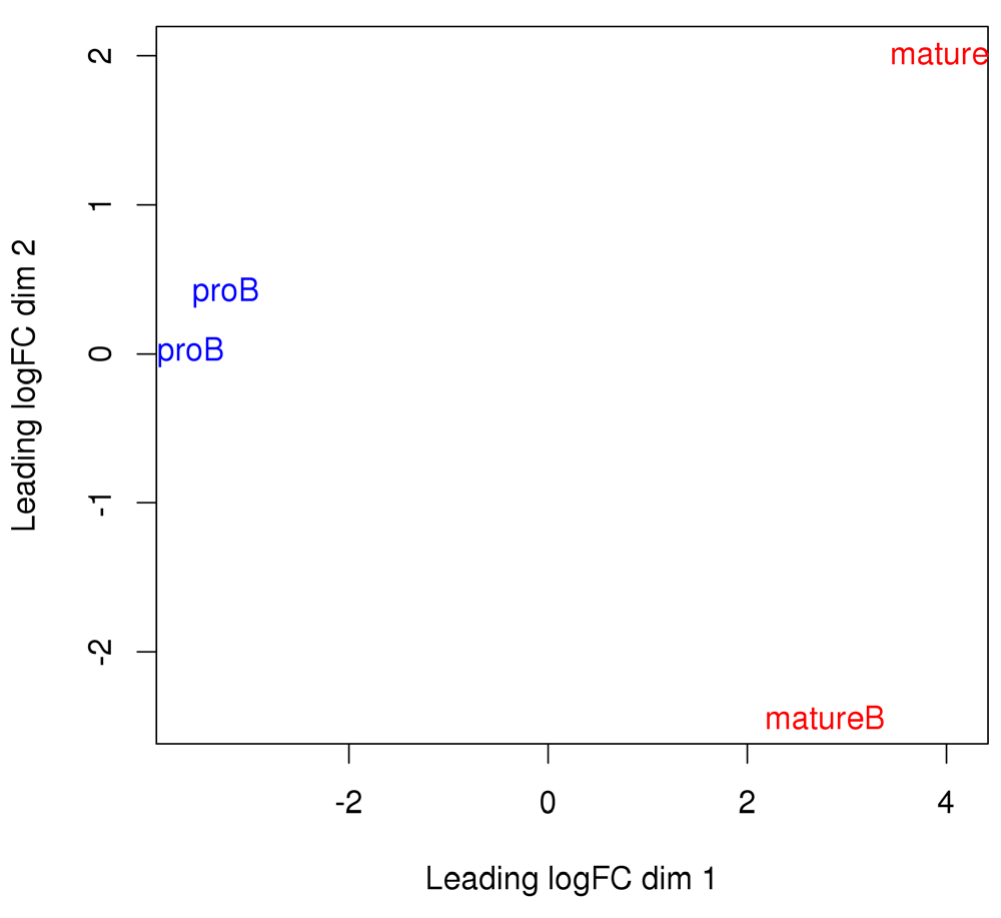
MDS plot with two dimensions for all libraries in the H3K9ac data set. Libraries are labelled and coloured according to the cell type.

***Examining the data with MDS plots.*** Multi-dimensional scaling (MDS) plots can be used to examine the similarities between libraries. The distance between a pair of libraries on this plot represents the overall log-fold change between those libraries. Ideally, replicates should cluster together while samples from different conditions should be separate. In Figure 7, strong separation in the first dimension is observed between libraries from different cell types. This indicates that significant differences are likely to be present between cell types in this data set.



                        plotMDS
                        (norm.adjc, 
                        labels=
                        celltype,
     
                        col=c
                        (
                        "red"
                        , 
                        "blue"
                        )[
                        as.integer
                        (celltype)])
                    


### Testing for DB and controlling the FDR

***Testing for DB with QL F-tests.*** Each window is tested for significant differences between cell types using the QL F-test ( Lund *et al.*, 2012). This is superior to the likelihood ratio test that is typically used for GLMs, as the QL F-test accounts for the uncertainity in dispersion estimation. One *p*-value is produced for each window, representing the evidence against the null hypothesis (i.e., that no DB is present in the window). For this analysis, the comparison is parametrized such that the reported log-fold change for each window represents that of the coverage in pro-B cells over their mature B counterparts.



                        contrast <- 
                        makeContrasts
                        (proB-matureB, 
                        levels=
                        design)
res <- 
                        glmQLFTest
                        (fit, 
                        contrast=
                        contrast)

                        head
                        (res$table)
                    




                        ##	 logFC	   logCPM	        F	    PValue
## 1 0.8071199	0.3987193	0.9350894	0.34292110
## 2 0.7892698	0.3531386	0.8977361	0.35257125
## 3 2.0508458	0.5770295	5.3124205	0.02987028
## 4 1.1952436	0.8317769	2.6800790	0.11429478
## 5 0.9751114	0.9868770	2.0577431	0.16397982
## 6 0.6472745	1.2487216	1.0906847	0.30643720
                    


***Controlling the FDR across regions.*** One might attempt to control the FDR by applying the Benjamini-Hochberg (BH) method to the window-level *p*-values ( Benjamini & Hochberg, 1995). However, the features of interest are not windows, but the genomic regions that they represent. Control of the FDR across windows does not guarantee control of the FDR across regions ( Lun & Smyth, 2014). The latter is arguably more relevant for the final interpretation of the results.

Control of the region-level FDR can be provided by aggregating windows into regions and combining the *p*-values. Here, adjacent windows less than 100 bp apart are aggregated into clusters. Each cluster represents a genomic region. Smaller values of tol allow distinct marking events to kept separate, while larger values provide a broader perspective, e.g., by considering adjacent co-regulated sites as a single entity. Chaining effects are mitigated by setting a maximum cluster width of 5 kbp.



                        merged <- 
                        mergeWindows
                        (
                        rowRanges
                        (filtered.data), 
                        tol=
                        100
                        , 
                        max.width=
                        5000
                        )
                    


A combined *p*-value is computed for each cluster using the method of Simes (1986), based on the *p*-values of the constituent windows. This represents the evidence against the global null hypothesis for each cluster, i.e., that no DB exists in any of its windows. Rejection of this global null indicates that the cluster (and the region that it represents) contains DB. Applying the BH method to the combined *p*-values allows the region-level FDR to be controlled.



                        tabcom <- 
                        combineTests
                        (merged$id, res$table)

                        head
                        (tabcom)
                    




                        ##   nWindows  logFC.up  logFC.down     PValue        FDR
## 1  	    2	      2		  0 0.35257125 0.48433052
## 2  	   24	     10		  0 0.03965980 0.09329214
## 3  	    8	      1		  3 0.38231836 0.51205966
## 4  	   11	      1		  2 0.84578866 0.91987081
## 5  	   36	     14		  6 0.01481647 0.04558244
## 6  	   18	      7		  9 0.00671594 0.02656732
                    


Each row of the output table contains the statistics for a single cluster, including the combined *p*-value before and after the BH correction. The nWindows field describes the total number of windows in the cluster. The logFC.up and logFC.down fields describe the number of windows with a log-fold change above 0.5 or below -0.5 in each cluster, respectively. This can be used to determine the direction of DB in each cluster.

***Examining the scope and direction of DB.*** The total number of DB regions at a FDR of 5% can be easily calculated.



                        is.sig <- tabcom$FDR <= 
                        0.05

                        summary
                        (is.sig)
                    




                        ##    Mode   FALSE    TRUE   NA’s
## logical   26121   13402      0
                    


Determining the direction of DB is more complicated, as clusters could potentially contain windows that are changing in opposite directions. One approach is to define the direction based on the number of windows changing in each direction, as described above. Another approach is to use the log-fold change of the most significant window as a proxy for the log-fold change of the cluster. This is generally satisfactory, though it will not capture multiple changes in opposite directions. It also tends to overstate the change in each cluster.



                        tabbest <- 
                        getBestTest
                        (merged$id, res$table)

                        head
                        (tabbest)
                    




                        ##   best      logFC	logCPM	        F     PValue        FDR
## 1    1  0.8071199 0.3987193  0.9350894 0.68584219 0.89635068
## 2   14  6.4894914 0.7814903 12.3651181 0.04305271 0.10940477
## 3   29 -0.8951569 1.4182105  3.1716524 0.70058621 0.91053977
## 4   42 -0.9100013 0.9724194  2.4590005 1.00000000 1.00000000
## 5   64  6.5014465 0.7867585 14.3069870 0.03337001 0.09138600
## 6   88  6.5134616 0.7920288 15.6865615 0.01067998 0.04156789
                    


In the above table, each row contains the statistics for each cluster. Of interest are the best and logFC fields. The former is the index of the window that is the most significant in each cluster, while the latter is the log-fold change of that window. This can be used to obtain a summary of the direction of DB across all clusters/regions.



                        is.sig.pos <- (tabbest$logFC > 
                        0
                        )[is.sig]

                        summary
                        (is.sig.pos)
                    




                        ##    Mode   FALSE    TRUE   NA’s
## logical    8137    5265      0
                    


### Saving results to file

Results can be saved to file prior to further manipulation. One approach is to store all statistics in the metadata of a GRanges object. This is useful as it keeps the statistics and coordinates together for each cluster, avoiding problems with synchronization in downstream steps. The midpoint and log-fold change of the best window are also stored.



                        out.ranges <- merged$region

                        elementMetadata
                        (out.ranges) <- 
                        data.frame
                        (tabcom,
   
                        best.pos=mid
                        (
                        ranges
                        (
                        rowRanges
                        (filtered.data[tabbest$best]))),
   
                        best.logFC=
                        tabbest$logFC)

                        saveRDS
                        (
                        file=
                        "h3k9ac_results.rds"
                        , out.ranges)
                    


For input into other programs like genome browsers, results can be saved in a more conventional format. Here, coordinates of DB regions are saved in BED format via *rtracklayer*, using a log-transformed FDR as the score.



                        simplified <- out.ranges[is.sig]
simplified$score <- -
                        10
                        *
                        log10
                        (simplified$FDR)

                        export
                        (
                        con=
                        "h3k9ac_results.bed"
                        , 
                        object=
                        simplified)
                    


Saving the RangedSummarizedExperiment objects is also recommended. This avoids the need to re-run the time-consuming read counting steps if parts of the analysis need to be repeated. Similarly, the DGEList object is saved so that the *edgeR* statistics can be easily recovered.



                        save
                        (
                        file=
                        "h3k9ac_objects.Rda"
                        , win.data, bins, y)
                    


## Interpreting the DB results

### Adding gene-centric annotation

***Using the detailRanges function.*** *csaw* provides its own annotation function, detailRanges. This identifies all genic features overlapping each region and reports them in a compact string form. Briefly, features are reported as SYMBOL|EXONS|STRAND where SYMBOL represents the gene symbol, EXONS lists the overlapping exons ( 0 for promoters, I for introns), and STRAND reports the strand. Multiple overlapping features for different genes are separated by commas within the string for each region.



                        library
                        (org.Mm.eg.db)

                        library
                        (TxDb.Mmusculus.UCSC.mm10.knownGene)
anno <- 
                        detailRanges
                        (out.ranges, 
                        orgdb=
                        org.Mm.eg.db,
     
                        txdb=
                        TxDb.Mmusculus.UCSC.mm10.knownGene)

                        head
                        (anno$overlap)
                    




                        ## [1] "Mrpl15|5|-"	"Mrpl15|0-1|-"	"Lypla1|0|+"	"Lypla1|0,2|+"
## [5] "Tcea1|0-2|+"	"Atp6v1h|0-1|+"
                    


Annotated features that flank the region of interest are also reported. The description for each feature is formatted as described above but with an extra [DISTANCE] field, representing the distance (in base pairs) between that feature and the region. By default, only flanking features within 5 kbp of each region are considered.



                        head
                        (anno$left)
                    




                        ## [1] "Mrpl15|6|-[935]"  "Mrpl15|2-3|-[896]" ""
## [4] "Lypla1|1|+[19]"	  ""		      ""
                    




                        head
                        (anno$right)
                    




                        ## [1] "Mrpl15|4|-[1875]"  ""		"Lypla1|1-2|+[143]"
## [4] ""		   ""	        "Atp6v1h|2|+[517]"
                    


The annotation for each region can then be stored in metadata of the GRanges object. The compact string form is useful for human interpretation, as it allows rapid examination of all genic features neighbouring each region.



                        meta <- 
                        elementMetadata
                        (out.ranges)

                        elementMetadata
                        (out.ranges) <- 
                        data.frame
                        (meta, anno)
                    


***Using the ChIPpeakAnno package.*** As its name suggests, the *ChIPpeakAnno* package is designed to annotate peaks from ChIP-seq experiments ( Zhu *et al.*, 2010). A GRanges object containing all regions of interest is supplied to the relevant function after removing all previous metadata fields to reduce clutter. The gene closest to each region is then reported. Gene coordinates are taken from the NCBI mouse 38 annotation, which is roughly equivalent to the annotation in the mm10 genome build.



                        library
                        (ChIPpeakAnno)

                        data
                        (TSS.mouse.GRCm38)
minimal <- out.ranges

                        elementMetadata
                        (minimal) <- 
                        NULL

                        anno.regions <- 
                        annotatePeakInBatch
                        (minimal, 
                        AnnotationData=
                        TSS.mouse.GRCm38)

                        colnames
                        (
                        elementMetadata
                        (anno.regions))
                    




                        ## [1] "peak"                     "feature"
## [3] "start_position"           "end_position"
## [5] "feature_strand"           "insideFeature"
## [7] "distancetoFeature"        "shortestDistance"
## [9] "fromOverlappingOrNearest"
                    


Alternatively, identification of all overlapping features within, say, 5 kbp can be achieved by setting maxgap=5000 and output="overlapping" in annotatePeakInBatch. This will report each overlapping feature in a separate entry of the returned GRanges object, i.e., each input region may have multiple output values. In contrast, detailRanges will report all overlapping features for a region as a single string, i.e., each input region has one output value. Which is preferable depends on the purpose of the annotation – the detailRanges output is more convenient for direct annotation of a DB list, while the annotatePeakInBatch output contains more information and is more convenient for further manipulation.

***Reporting gene-based results.*** Another approach to annotation is to flip the problem around, such that DB statistics are reported directly for features of interest like genes. This is more convenient when the DB analysis needs to be integrated with, e.g., DE analyses of matching RNA-seq data. In the code below, promoter coordinates are obtained by running detailRanges without specifying any regions. All windows overlapping each promoter are defined as a cluster, and DB statistics are computed as previously described for each cluster/promoter. This directly yields DB results for annotated features, along with some NA values representing promoters that have no overlapping windows (these are filtered out in the code below for demonstration purposes).



                        anno.ranges  <- 
                        detailRanges
                        (
                        orgdb=
                        org.Mm.eg.db,
     
                        txdb=
                        TxDb.Mmusculus.UCSC.mm10.knownGene)
promoters  <- anno.ranges[anno.ranges$exon==0L]
olap <- 
                        findOverlaps
                        (promoters, 
                        rowRanges
                        (filtered.data))
tabprom <- 
                        combineOverlaps
                        (olap, res$table)

                        head
                        (
                        data.frame
                        (
                        Gene=
                        promoters$symbol,  tabprom)[!
                        is.na
                        (tabprom$PValue),])
                    




                        ##	 Gene nWindows logFC.up logFC.down	   PValue	   FDR
## 6  Ldlrap1	    19	     11		 0 0.224741404877 0.2707231899
## 7     Mdn1	    29	     12		11 0.000004447727 0.0001346831
## 8    Pydc3	     8        0		 6 0.051183399851 0.0781862855
## 9   Wfdc17	     6	      0		 6 0.000069604922 0.0008739367
## 10  Mfap1b	    19	      1		10 0.107116609335 0.1441133306
## 13 Gm15772	    30	     12		 7 0.085543435687 0.1193045223
                    


Note that this strategy is distinct from counting reads across promoters. Using promoter-level counts would not provide enough spatial resolution to detect sharp binding events that only occur in a subinterval of the promoter. In particular, detection may be compromised by non-specific background or the presence of multiple opposing DB events in the same promoter. Combining window-level statistics is preferable as resolution is maintained for optimal performance.

### Visualizing DB results

***Overview.*** Here, the *Gviz* package is used to visualize read coverage across the data set at regions of interest. Coverage in each BAM file will be represented by a single track. Several additional tracks will also be included in each plot. One is the genome axis track, to display the genomic coordinates across the plotted region. The other is the annotation track containing gene models, with gene IDs replaced by symbols (where possible) for easier reading.



                        library
                        (Gviz)
gax <- 
                        GenomeAxisTrack
                        (
                        col=
                        "black"
                        , 
                        fontsize=
                        15
                        , 
                        size=
                        2
                        )
greg <- 
                        GeneRegionTrack
                        (TxDb.Mmusculus.UCSC.mm10.knownGene, 
                        showId=
                        TRUE
                        ,
     
                        geneSymbol=
                        TRUE
                        , 
                        name=
                        ""
                        , 
                        background.title=
                        "transparent"
                        )
symbols <- 
                        unlist
                        (
                        mapIds
                        (org.Mm.eg.db, 
                        gene
                        (greg), 
                        "SYMBOL"
                        ,
     
                        "ENTREZID"
                        , 
                        multiVals = 
                        "first"
                        ))

                        symbol
                        (greg) <- symbols[
                        gene
                        (greg)]
                    


**Figure 8.**
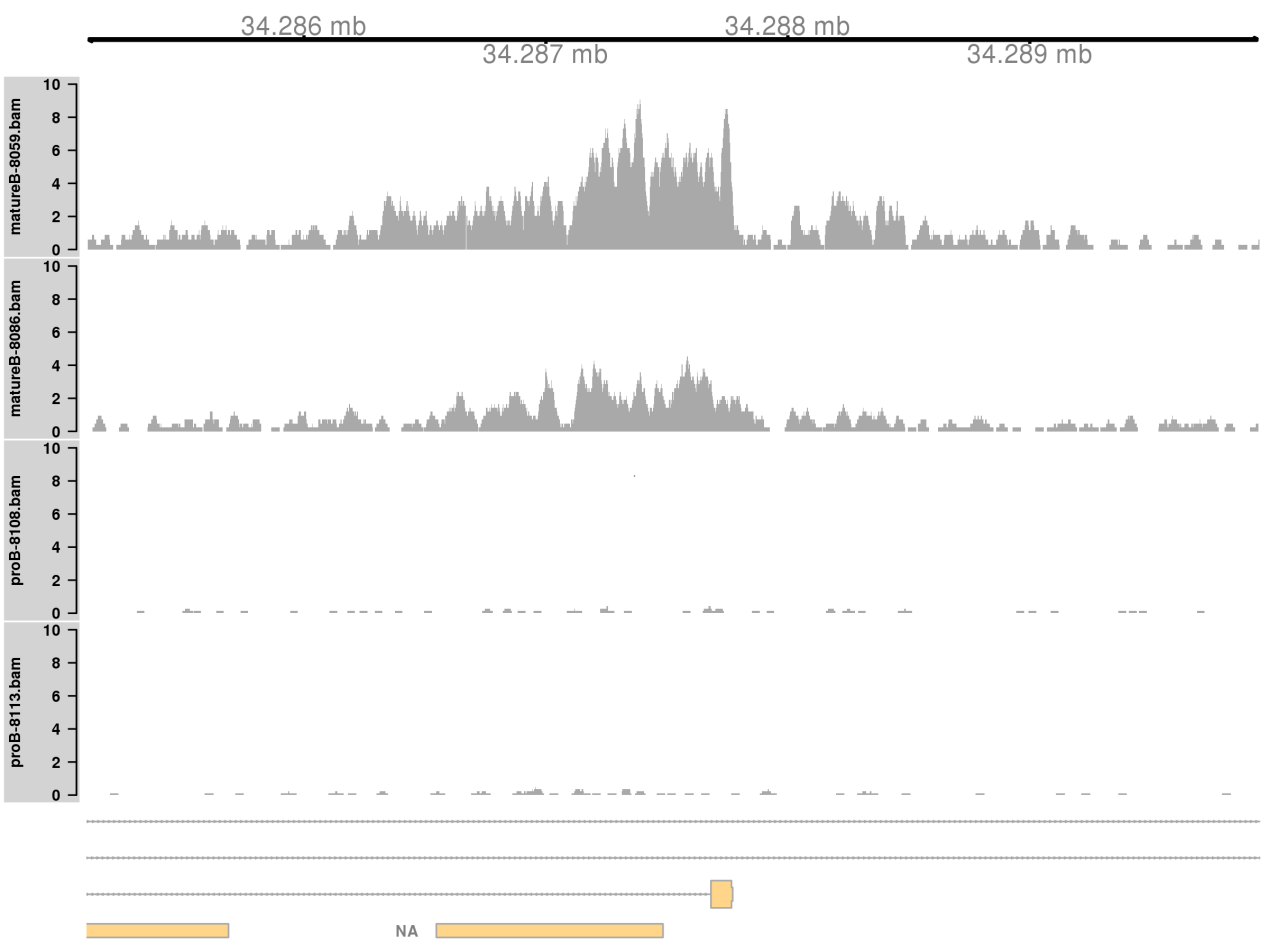
Coverage tracks for a simple DB event between pro-B and mature B cells, across a broad region in the H3K9ac data set. Read coverage for each library is shown as a per-million value at each base.



***Simple DB across a broad region.*** To begin with, the top-ranking DB region will be visualized. This represents a simple DB event where the entire region changes in one direction ( Figure 8). Specifically, it represents an increase in H3K9ac marking at the *H2-Aa* locus. This is consistent with the expected biology – H3K9ac is a mark of active gene expression ( Karmodiya *et al.*, 2012) and MHCII components are upregulated in mature B cells ( Hoffmann *et al.*, 2002).

                        o <- 
                        order
                        (out.ranges$PValue)
cur.region <- out.ranges[o[
                        1
                        ]]
cur.region
                    




                        ## GRanges object with 1 range and 10 metadata columns:
##	 seqnames	        ranges strand |	 nWindows  logFC.up
##	    <Rle>	     <IRanges>  <Rle> | <integer> <integer>
##   [1]    chr17 [34285101, 34289950]	    * |	       94	  0
##	 logFC.down		    PValue		 FDR   best.pos
##	  <integer>		 <numeric>	   <numeric>  <integer>
##   [1]	 94 0.00000000000004471753 0.000000001195279   34287575
##	 best.logFC			      overlap		   left
##	  <numeric>			     <factor>	       <factor>
##   [1]  -7.176575 H2-Aa|0-1|-,H2-Eb1|I|+,Notch4|I|+ H2-Aa|2-6|-[278]
##	    right
##	 <factor>
##   [1]
##   -------
##   seqinfo: 21 sequences from an unspecified genome
                    


One track is plotted for each library, in addition to the coordinate and annotation tracks. Coverage is plotted in terms of sequencing depth-per-million at each base. This corrects for differences in library sizes between tracks.



                        collected <- 
                        list
                        ()
lib.sizes <- filtered.data$totals/
                        1e6

                        for (i in 
                        1
                        :
                        length
                        (bam.files)) {
    reads <- 
                        extractReads
                        (
                        bam.file=
                        bam.files[i], cur.region, 
                        param=
                        param)
    cov <- 
                        as
                        (
                        coverage
                        (reads)/lib.sizes[i], 
                        "GRanges"
                        )
    collected[[i]] <- 
                        DataTrack
                        (cov, 
                        type=
                        "histogram"
                        , 
                        lwd=
                        0
                        , 
                        ylim=c
                        (
                        0
                        ,
                        10
                        ),
         
                        name=
                        bam.files[i],  
                        col.axis=
                        "black"
                        , 
                        col.title=
                        "black"
                        ,
         
                        fill=
                        "darkgray"
                        , 
                        col.histogram=
                        NA
                        )
}

                        plotTracks
                        (
                        c
                        (gax, collected, greg), 
                        chromosome=as.character
                        (
                        seqnames
                        (cur.region)),
     
                        from=start
                        (cur.region), 
                        to=end
                        (cur.region))
                    


***Complex DB across a broad region.*** Complex DB refers to situations where multiple DB events are occurring within the same enriched region. These are identified as those clusters that contain windows changing in both directions. Here, the second-ranking complex cluster is selected for visualization (the top-ranking complex cluster is adjacent to the region used in the previous example, so another region is chosen for some variety).



                        complex <- out.ranges$logFC.up > 
                        0 
                        & out.ranges$logFC.down > 
                        0

                        cur.region <- out.ranges[o[complex[o]][
                        2
                        ]]
cur.region
                    




                        ## GRanges object with 1 range and 10 metadata columns:
##	 seqnames	          ranges strand |  nWindows  logFC.up
##	    <Rle>	       <IRanges>  <Rle> | <integer> <integer>
##   [1]     chr5 [122987201, 122991450]      * |        83 	   17
##	 logFC.down		PValue		   FDR  best.pos best.logFC
##	  <integer>  	     <numeric>	     <numeric> <integer>  <numeric>
##   [1]	 43 0.0000000002201102 0.0000001962277 122990925  -5.466918
##	              	         overlap	      left
##	              	        <factor>	  <factor>
##   [1] A930024E05Rik|0-1|+,Kdm2b|0-3|- Kdm2b|4-5|-[2661]
##	    		   right
##	    		<factor>
##   [1] A930024E05Rik|2|+[2913]
##   -------
##   seqinfo: 21 sequences from an unspecified genome
                    


**Figure 9.**
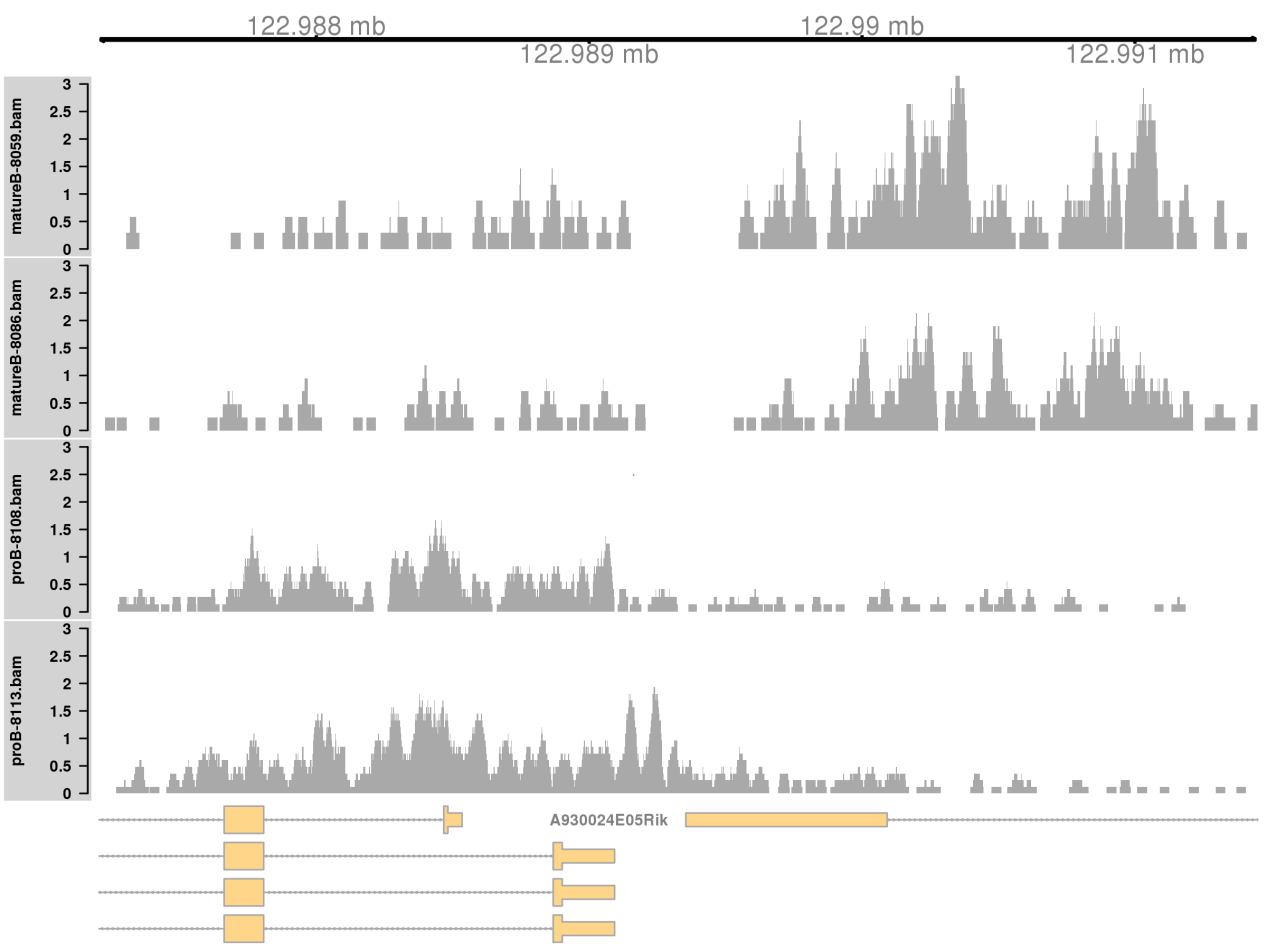
Coverage tracks for a complex DB event in the H3K9ac data set, shown as per-million values.

This region contains a bidirectional promoter where different genes are marked in the different cell types ( Figure 9). Upon differentiation to mature B cells, loss of marking in one part of the region is balanced by a gain in marking in another part of the region. This represents a complex DB event that would not be detected if reads were counted across the entire region.



                        collected <- 
                        list
                        ()

                        for (i in 
                        1
                        :
                        length
                        (bam.files)) {
    reads <- 
                        extractReads
                        (
                        bam.file=
                        bam.files[i], cur.region, 
                        param=
                        param)
    cov <- 
                        as
                        (
                        coverage
                        (reads)/lib.sizes[i], 
                        "GRanges"
                        )
    collected[[i]] <- 
                        DataTrack
                        (cov, 
                        type=
                        "histogram"
                        , 
                        lwd=
                        0
                        , 
                        ylim=c
                        (
                        0
                        ,
                        3
                        ),
         
                        name=
                        bam.files[i],  
                        col.axis=
                        "black"
                        , 
                        col.title=
                        "black"
                        , 
                        fill=
                        "darkgray"
                        , 
                        col.histogram=
                        NA
                        )
}

                        plotTracks
                        (
                        c
                        (gax, collected, greg), 
                        chromosome=as.character
                        (
                        seqnames
                        (cur.region)),
     
                        from=start
                        (cur.region), 
                        to=end
                        (cur.region))
                    


***Simple DB across a small region.*** Both of the examples above involve differential marking within broad regions spanning several kilobases. This is consistent with changes in the marking profile across a large number of nucleosomes. However, H3K9ac marking can also be concentrated into small regions, involving only a few nucleosomes. *csaw* is equally capable of detecting sharp DB within these small regions. This can be demonstrated by examining those clusters that contain a smaller number of windows.



                        sharp <- out.ranges$nWindows < 
                        20

                        cur.region <- out.ranges[o[sharp[o]][
                        1
                        ]]
cur.region
                    




                        ## GRanges object with 1 range and 10 metadata columns:
##	 seqnames	        ranges strand |	 nWindows  logFC.up
##	    <Rle>	     <IRanges>  <Rle> | <integer> <integer>
##   [1]    chr16 [36665551, 36666200]	    * |	       11	  0
##	 logFC.down		PValue		   FDR  best.pos best.logFC
##	  <integer>  	     <numeric>	     <numeric> <integer>  <numeric>
##   [1]	 11 0.0000000003412784 0.0000002593913  36665925  -4.887727
##	    overlap     left    right
##	   <factor> <factor> <factor>
##   [1] Cd86|0-1|-
##   -------
##   seqinfo: 21 sequences from an unspecified genome
                    


**Figure 10.**
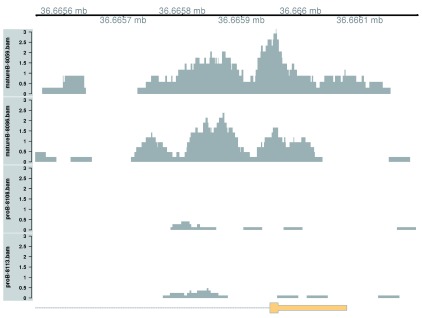
Coverage tracks for a sharp and simple DB event in the H3K9ac data set, shown as per-million values.



Marking is increased for mature B cells within a 500 bp region ( Figure 10), which is sharper than the changes in the previous two examples. This also coincides with the promoter of the *Cd86* gene. Again, this makes biological sense as CD86 is involved in regulating immunoglobulin production in activated B-cells ( Podojil & Sanders, 2003).

                        collected <- 
                        list
                        ()
for (i in 
                        1
                        :
                        length
                        (bam.files)) {
    reads <- 
                        extractReads
                        (
                        bam.file=
                        bam.files[i], cur.region, 
                        param=
                        param)
    cov <- 
                        as
                        (
                        coverage
                        (reads)/lib.sizes[i], 
                        "GRanges"
                        )
    collected[[i]] <- 
                        DataTrack
                        (cov, 
                        type=
                        "histogram"
                        , 
                        lwd=
                        0
                        , 
                        ylim=c
                        (
                        0
                        ,
                        3
                        ),
          
                        name=
                        bam.files[i], 
                        col.axis=
                        "black"
                        , 
                        col.title=
                        "black"
                        ,
          
                        fill=
                        "darkgray"
                        ,  
                        col.histogram=
                        NA
                        )
}

                        plotTracks
                        (
                        c
                        (gax, collected, greg), 
                        chromosome=as.character
                        (
                        seqnames
                        (cur.region)),
     
                        from=start
                        (cur.region), 
                        to=end
                        (cur.region))
                    


Note that the window size will determine whether sharp or broad events are preferentially detected. Larger windows provide more power to detect broad events (as the counts are higher), while smaller windows provide more resolution to detect sharp events. Optimal detection of all features can be obtained by performing analyses with multiple window sizes and consolidating the results, though – for brevity – this will not be described here. In general, smaller window sizes are preferred as strong DB events with sufficient coverage will always be detected. For larger windows, detection may be confounded by other events within the window that distort the log-fold change in the counts between conditions.

## Repeating the analysis for the CBP data

### Overview

A window-based DB analysis will be shown for transcription factor (TF) data, to complement the histone mark analysis above. This data set compares CBP binding between wild-type (WT) and CBP knock-out (KO) animals ( Kasper *et al.*, 2014). The aim is to use *csaw* and other Bioconductor packages to identify DB sites between genotypes. Most, if not all, of these sites should be increased in the WT, given that protein function should be compromised in the KO.

### Aligning reads from CBP libraries

Libraries are downloaded from the NCBI GEO data series GSE54453, using the SRA accessions listed below. The data set contains two biological replicates for each of the two genotypes. One file is available for each library, i.e., no technical replicates.



                        sra.numbers <- 
                        c
                        (
                        "SRR1145787"
                        , 
                        "SRR1145788"
                        , 
                        "SRR1145789"
                        , 
                        "SRR1145790"
                        )
genotype <- 
                        c
                        (
                        "wt"
                        , 
                        "wt"
                        , 
                        "ko"
                        , 
                        "ko"
                        )
all.sra <- 
                        paste0
                        (sra.numbers, 
                        ".sra"
                        )

                        data.frame
                        (
                        SRA=
                        all.sra, 
                        Condition=
                        genotype)
                    




                        ##		SRA Condition
## 1 SRR1145787.sra	   wt
## 2 SRR1145788.sra	   wt
## 3 SRR1145789.sra	   ko
## 4 SRR1145790.sra	   ko
                    


SRA files are unpacked to yield FASTQ files with the raw read sequences.



                        for (sra in all.sra) {
    code <- 
                        system
                        (
                        paste
                        (
                        "fastq-dump"
                        , sra))
    
                        stopifnot
                        (code==0L)
}
all.fastq <- 
                        paste0
                        (sra.numbers, 
                        ".fastq"
                        )
                    


Reads are aligned to the mm10 genome using *Rsubread*. Here, the default consensus threshold is used as the reads are longer (75 bp). A Phred offset of +64 is also used, instead of the default +33 used in the previous analysis.



                        bam.files <- 
                        paste0
                        (sra.numbers, 
                        ".bam"
                        )

                        align
                        (
                        index=
                        "index/mm10"
                        , 
                        readfile1=
                        all.fastq, 
                        type=
                        1
                        , 
                        phredOffset=
                        64
                        ,
     
                        input_format=
                        "FASTQ"
                        , 
                        output_file=
                        bam.files)
                    


Alignments in each BAM file are sorted by coordinate. Duplicate reads are marked, and the resulting files are indexed.



                        temp.bam <- 
                        "cbp_temp.bam"

                        temp.file <- 
                        "cbp_metric.txt"

                        temp.dir <- 
                        "cbp_working"

                        dir.create
                        (temp.dir)
for (bam in bam.files) {
    out <- 
                        suppressWarnings
                        (
                        sortBam
                        (bam, 
                        "cbp_temp"
                        ))
     
                        file.rename
                        (out, bam)
    code <- 
                        system
                        (
                        sprintf
                        (
                        "MarkDuplicates I=%s O=%s M=%s \\
        TMP_DIR=%s AS=true REMOVE_DUPLICATES=false \\
        VALIDATION_STRINGENCY=SILENT"
                        ,
        bam, temp.bam, temp.file, temp.dir))
     
                        stopifnot
                        (code==0L)
     
                        file.rename
                        (temp.bam, bam)
}

                        indexBam
                        (bam.files)
                    


Some mapping statistics can be reported as previously described. For brevity, the code will not be shown here, as it is identical to that used for the H3K9ac analysis.



                        ##		     Total   Mapped  Marked Prop.mapped Prop.marked
## SRR1145787.bam 28525952 24015041 2244935    84.18664    9.348037
## SRR1145788.bam 25514465 21288115 2062157    83.43547    9.686893
## SRR1145789.bam 34476967 28830024 2678297    83.62111    9.289958
## SRR1145790.bam 32624587 27067108 2912659    82.96537   10.760880
                    


### Detecting DB between genotypes for CBP

***Counting reads into windows.*** First, a readParam object is constructed to standardize the parameter settings in this analysis. The ENCODE blacklist is again used to remove reads in problematic regions. For consistency, the MAPQ threshold of 50 is also re-used here for removing poorly aligned reads. Lower thresholds (e.g., from 10 to 20) can be used for longer reads with more reliable mapping locations - though in practice, the majority of long read alignments reported by *Rsubread* tend to have very high or very low MAPQ scores, such that the exact choice of the MAPQ threshold is not a critical parameter.



                        param <- 
                        readParam
                        (
                        minq=
                        50
                        , 
                        discard=
                        blacklist)
                    


The average fragment length is estimated by maximizing the cross-correlation function, as previously described.



                        x <- 
                        correlateReads
                        (bam.files, 
                        param=reform
                        (param, 
                        dedup=
                        TRUE
                        ))
frag.len <- 
                        which.max
                        (x) - 
                        1

                        frag.len
                    




                        ## [1] 162
                    


Reads are then counted into sliding windows. For TF data analyses, smaller windows are necessary to capture sharp binding sites. A large window size will be suboptimal as the count for a particular site will be “contaminated” by non-specific background in the neighbouring regions. In this case, a window size of 10 bp is used.



                        win.data <- 
                        windowCounts
                        (bam.files, 
                        param=
                        param, 
                        width=
                        10
                        , 
                        ext=
                        frag.len)
win.data
                    




                        ## class: RangedSummarizedExperiment
## dim: 9127613 4
## metadata(4): spacing width shift final.ext
## assays(1): counts
## rownames: NULL
## rowRanges metadata column names(0):
## colnames: NULL
## colData names(4): bam.files totals ext param
                    


The default spacing of 50 bp is also used here. This may seem inappropriate, given that the windows are only 10 bp. However, reads lying in the interval between adjacent windows will still be counted into several windows. This is because reads are extended to the value of frag.len, which is substantially larger than the 50 bp spacing. Again, smaller spacings can be used but will provide little benefit, given that each extended read already overlaps multiple windows.

***Normalization for composition biases.*** Composition biases are introduced when the amount of DB in each condition is unbalanced ( Lun & Smyth, 2014; Robinson & Oshlack, 2010). More binding in one condition means that more reads are sequenced at the binding sites, leaving fewer reads for the rest of the genome. This suppresses the genomic coverage at non-DB sites, resulting in spurious differences between libraries. To remove this bias, reads are counted into large genomic bins. Most bins are assumed to represent non-DB background regions. Any systematic differences in the coverage of those bins is attributed to composition bias and is normalized out. Specifically, the TMM method ( Robinson & Oshlack, 2010) is applied to compute normalization factors from the bin counts. These factors can then be applied to the DB analysis with the window counts.



                        bins <- 
                        windowCounts
                        (bam.files, 
                        bin=
                        TRUE
                        , 
                        width=
                        10000
                        , 
                        param=
                        param)
normfacs <- 
                        normOffsets
                        (bins)
normfacs
                    




                        ## [1] 1.011851 0.908138 1.044806 1.041588
                    


**Figure 11.**
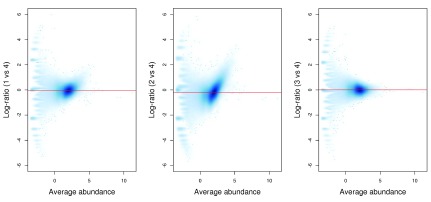
Mean-difference plots for the bin counts, comparing library 4 to all other libraries. The red line represents the log-ratio of the normalization factors between libraries.

The effect of normalization can be visualized with some mean-difference plots between pairs of libraries ( Figure 11). The dense cloud in each plot represents the majority of bins in the genome. These are assumed to mostly contain background regions. A non-zero log-fold change for these bins indicates that composition bias is present between libraries. The red line represents the log-ratio of normalization factors and passes through the centre of the cloud in each plot, indicating that the bias has been successfully identified and removed.



                        y.bin <- 
                        asDGEList
                        (bins)
bin.ab <- 
                        aveLogCPM
                        (y.bin)

                        adjc <- 
                        cpm
                        (y.bin, 
                        log=
                        TRUE
                        )

                        par
                        (
                        cex.lab=
                        1.5
                        , 
                        mfrow=c
                        (
                        1
                        ,
                        3
                        ))

                        smoothScatter
                        (bin.ab, adjc[,
                        1
                        ]-adjc[,
                        4
                        ], 
                        ylim=c
                        (-
                        6
                        , 
                        6
                        ),
     
                        xlab=
                        "Average abundance"
                        , 
                        ylab=
                        "Log-ratio (1 vs 4)"
                        )

                        abline
                        (
                        h=log2
                        (normfacs[
                        1
                        ]/normfacs[
                        4
                        ]), 
                        col=
                        "red"
                        )

                        smoothScatter
                        (bin.ab, adjc[,
                        2
                        ]-adjc[,
                        4
                        ], 
                        ylim=c
                        (-
                        6
                        , 
                        6
                        ),
     
                        xlab=
                        "Average abundance"
                        , 
                        ylab=
                        "Log-ratio (2 vs 4)"
                        )

                        abline
                        (
                        h=log2
                        (normfacs[
                        2
                        ]/normfacs[
                        4
                        ]), 
                        col=
                        "red"
                        )

                        smoothScatter
                        (bin.ab, adjc[,
                        3
                        ]-adjc[,
                        4
                        ], 
                        ylim=c
                        (-
                        6
                        , 
                        6
                        ),
     
                        xlab=
                        "Average abundance"
                        , 
                        ylab=
                        "Log-ratio (3 vs 4)"
                        )

                        abline
                        (
                        h=log2
                        (normfacs[
                        3
                        ]/normfacs[
                        4
                        ]), 
                        col=
                        "red"
                        )
                    


Note that this normalization strategy is quite different from that in the H3K9ac analysis. Here, systematic DB in one direction is expected between conditions, given that CBP function is lost in the KO genotype. This means that the assumption of a non-DB majority (required for non-linear normalization of the H3K9ac data) is not valid. No such assumption is made by the binned-TMM approach described above, which makes it more appropriate for use in the CBP analysis.

***Filtering of low-abundance windows.*** Removal of low-abundance windows is performed as previously described. The majority of windows in background regions are filtered out upon applying a modest fold-change threshold. This leaves a small set of relevant windows for further analysis.



                        filter.stat <- 
                        filterWindows
                        (win.data, bins, 
                        type=
                        "global"
                        )
min.fc <- 
                        3

                        keep <- filter.stat$filter > 
                        log2
                        (min.fc)

                        summary
                        (keep)
                    




                        ##     Mode   FALSE    TRUE   NA’s
##  logical 8862335  265278      0
                    




                        filtered.data <- win.data[keep,]
                    


It should be noted that the 10 kbp bins are used here for filtering, while smaller 2 kbp bins were used in the corresponding step for the H3K9ac analysis. This is purely for convenience – the 10 kbp counts for this data set were previously loaded for normalization, and can be re-used during filtering to save time. Changes in bin size will have little impact on the results, so long as the bins (and their counts) are large enough for precise estimation of the background abundance. While smaller bins provide greater spatial resolution, this is irrelevant for quantifying coverage in large background regions that span most of the genome.

***Statistical modelling of biological variability.*** Counts for each window are modelled using *edgeR* as previously described. First, a design matrix needs to be constructed.



                        genotype <- 
                        factor
                        (genotype)
design <- 
                        model.matrix
                        (~
                        0
                        +genotype)

                        colnames
                        (design) <- 
                        levels
                        (genotype)
design
                    




                        ##   ko wt
## 1  0  1
## 2  0  1
## 3  1  0
## 4  1  0
## attr(,"assign")
## [1] 1 1
## attr(,"contrasts")
## attr(,"contrasts")$genotype
## [1] "contr.treatment"
                    


Estimation of the NB and QL dispersions is then performed. The estimated NB dispersions are substantially larger than those observed in the H3K9ac data set. In addition, the estimated prior d.f. is infinite.



                        y <- 
                        asDGEList
                        (filtered.data, 
                        norm.factors=
                        normfacs)
y <- 
                        estimateDisp
                        (y, design)

                        summary
                        (y$trended.dispersion)
                    




                        ##    Min. 1st Qu.  Median    Mean 3rd Qu.    Max.
##  0.1376  0.1641  0.1835  0.1895  0.2127  0.2572
                    




                        fit <- 
                        glmQLFit
                        (
                            y, design, 
                        robust=
                        TRUE
                        )

                        summary
                        (fit$df.prior)
                    




                        ##     Min. 1st Qu.  Median    Mean 3rd Qu.    Max.
##	Inf	Inf	Inf	Inf	Inf	Inf
                    


**Figure 12.**
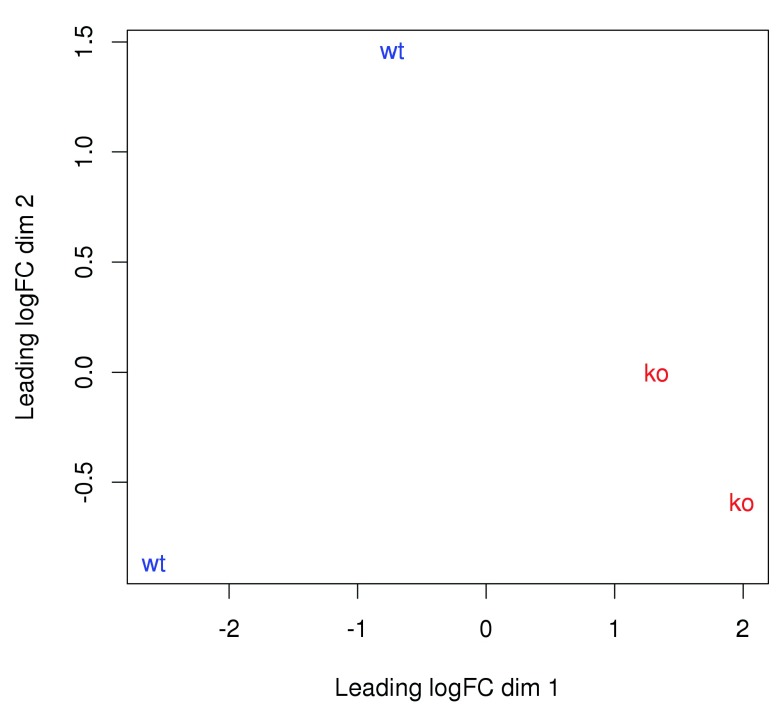
MDS plot with two dimensions for all libraries in the CBP data set. Libraries are labelled and coloured according to the genotype. A larger top set of windows was used to improve the visualization of the genome-wide differences between the WT libraries.



These statistics are consistent with the presence of a batch effect between replicates. The dispersions for all windows are inflated to a similarly large value by the batch effect, resulting in low variability in the dispersions across windows. This is illustrated in Figure 12 where the WT libraries are clearly separated in both dimensions of the MDS plot. In particular, separation of replicates on the first dimension is indicative of a systematic difference of size comparable to that between genotypes.

                        plotMDS(
                        cpm
                        (y, 
                        log=
                        TRUE
                        ), 
                        top=
                        10000
                        , 
                        labels=
                        genotype,
    
                        col=c
                        (
                        "red"
                        , 
                        "blue"
                        )[
                        as.integer
                        (genotype)])
                    


The presence of a large batch effect between replicates is not ideal. Nonetheless, the DB analysis can proceed, albeit with some loss of power due to the inflated NB dispersions.

***Testing for DB.*** DB windows are identified using the QL F-test. Windows are clustered into regions, and the region-level FDR is controlled using Simes’ method. All significant regions have increased CBP binding in the WT genotype. This is expected, given that protein function should be lost in the KO genotype.



                        contrast <- 
                        makeContrasts
                        (wt-ko, 
                        levels=
                        design)
res <- 
                        glmQLFTest
                        (fit, 
                        contrast=
                        contrast)
merged <- 
                        mergeWindows
                        (
                        rowRanges
                        (filtered.data), 
                        tol=
                        100
                        , 
                        max.width=
                        5000
                        )

                        tabcom <- 
                        combineTests
                        (merged$id, res$table)

                        tabbest <- 
                        getBestTest
                        (merged$id, res$table)
is.sig <- tabcom$FDR <= 
                        0.05

                        summary
                        (is.sig)
                    




                        ##    Mode   FALSE    TRUE   NA’s
## logical   55444    1969      0
                    




                        is.sig.pos <- (tabbest$logFC > 
                        0
                        )[is.sig]

                        summary
                        (is.sig.pos)
                    




                        ##    Mode    TRUE   NA’s
## logical    1969      0
                    


These results can be saved to file, as previously described. Key objects are also saved for convenience.



                        out.ranges <- merged$region

                        elementMetadata
                        (out.ranges) <- 
                        data.frame
                        (tabcom,
     
                        best.pos=mid
                        (
                        ranges
                        (
                        rowRanges
                        (filtered.data[tabbest$best]))),
     
                        best.logFC=
                        tabbest$logFC)

                        saveRDS
                        (
                        file=
                        "cbp_results.rds"
                        , out.ranges)

                        save
                        (
                        file=
                        "cbp_objects.Rda"
                        , win.data, bins, y)
                    


### Annotation and visualization

Annotation is added using the detailRanges function, as previously described.



                        anno <- 
                        detailRanges
                        (out.ranges, 
                        orgdb=
                        org.Mm.eg.db,
	      
                        txdb=
                        TxDb.Mmusculus.UCSC.mm10.knownGene)
meta <- 
                        elementMetadata
                        (out.ranges)

                        elementMetadata
                        (out.ranges) <- 
                        data.frame
                        (meta, anno)
                    


The top-ranked DB event will be visualized here. This corresponds to a simple DB event, as all windows are changing in the same direction, i.e., up in the WT. The binding region is also quite small relative to some of the H3K9ac examples, consistent with sharp TF binding to a specific recognition site.



                        o <- 
                        order
                        (out.ranges$PValue)
cur.region <- out.ranges[o[
                        1
                        ]]
cur.region
                    




                        ## GRanges object with 1 range and 10 metadata columns:
##	 seqnames	        ranges strand |	 nWindows  logFC.up
##	    <Rle>	     <IRanges>  <Rle> | <integer> <integer>
##   [1]    chr16 [70313851, 70314860]	    * |	       21	 21
##	 logFC.down	     PValue	   FDR  best.pos best.logFC
##	  <integer>	  <numeric>  <numeric> <integer>  <numeric>
##   [1]	  0 0.0000001802112 0.00348259	70314405   5.273053
##	    overlap	left	right
##	   <factor> <factor> <factor>
##   [1] Gbe1|0-1|+
##   -------
##   seqinfo: 66 sequences from an unspecified genome
                    


**Figure 13.**
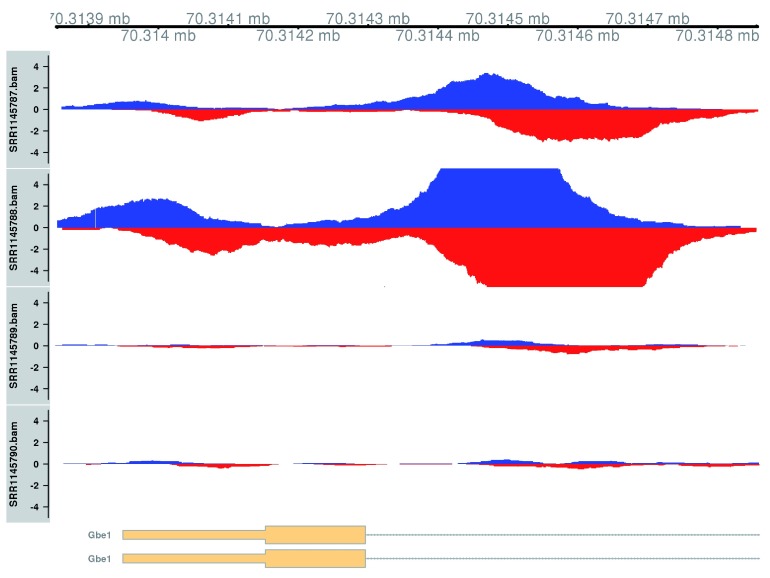
Coverage tracks for TF binding sites that are differentially bound in the WT (top two tracks) against the KO (last two tracks). Blue and red tracks represent forward- and reverse-strand coverage, respectively, on a per-million scale (capped at 5 in SRR1145788, for visibility).



Plotting is performed using two tracks for each library – one for the forward-strand coverage, another for the reverse-strand coverage. This allows visualization of the strand bimodality that is characteristic of genuine TF binding sites. In Figure 13, two adjacent sites are present at the *Gbe1* promoter, both of which exhibit increased binding in the WT genotype. Coverage is also substantially different between the WT replicates, consistent with the presence of a batch effect.

                        collected <- 
                        list
                        ()
lib.sizes <- filtered.data$totals/
                        1e6

                        for (i in 
                        1
                        :
                        length
                        (bam.files)) {
    reads <- 
                        extractReads
                        (
                        bam.file=
                        bam.files[i], cur.region, 
                        param=
                        param)
    
                        pcov <- 
                        as
                        (
                        coverage
                        (reads[
                        strand
                        (reads)==
                        "+"
                        ])/lib.sizes[i], 
                        "GRanges"
                        )
    ncov <- 
                        as
                        (
                        coverage
                        (reads[
                        strand
                        (reads)==
                        "-"
                        ])/-lib.sizes[i], 
                        "GRanges"
                        )
    ptrack <- 
                        DataTrack
                        (pcov, 
                        type=
                        "histogram"
                        ,  
                        lwd=
                        0
                        , 
                        ylim=c
                        (
                        -5
                        , 
                        5
                        ),
          
                        name=
                        bam.files[i], 
                        col.axis=
                        "black"
                        , 
                        col.title=
                        "black"
                        ,
          
                        fill=
                        "blue"
                        , 
                        col.histogram=
                        NA
                        )
    ntrack <- 
                        DataTrack
                        (ncov, 
                        type=
                        "histogram"
                        , 
                        lwd=
                        0
                        , 
                        ylim=c
                        (
                        -5
                        , 
                        5
                        ),
          
                        fill=
                        "red"
                        , 
                        col.histogram=
                        NA
                        )
    collected[[i]] <- 
                        OverlayTrack
                        (
                        trackList=list
                        (ptrack, ntrack))
}

                        plotTracks
                        (c
                        (gax, collected, greg), 
                        chromosome=as.character
                        (
                        seqnames
                        (cur.region)),
     
                        from=start
                        (cur.region), 
                        to=end
                        (cur.region))
                    


Note that that the gax and greg objects are the same as those used in the visualization of the H3k9ac data.

## Summary

This workflow describes the steps of a window-based DB analysis, from read alignment through to visualization of DB regions. All steps are performed within the R environment and mostly use functions from Bioconductor packages. In particular, the core of the workflow – the detection of DB regions – is based on a combination of *csaw* and *edgeR*. Analyses are shown for histone mark and TF data sets, with differences in parametrization that are appropriate to each data type. Readers are encouraged to apply the concepts and code presented in this article to their own data.

## Software availability

This workflow depends on various packages from version 3.2 of the Bioconductor project, running on *R* version 3.2.2 or higher. It requires a number of software packages, including *csaw*, *edgeR*, *Rsubread*, *Rsamtools*, *Gviz*, *rtracklayer* and *ChIPpeakAnno*. It also depends on the annotation packages *org.Mm.eg.db* and *TxDb.Mmusculus.UCSC.mm10.knownGene*. Version numbers for all packages used are shown below.



                    sessionInfo()
                




                    ## R version 3.2.2 Patched (2015-10-30 r69588)
## Platform: x86_64-pc-linux-gnu (64-bit)
## Running under: CentOS release 6.4 (Final)
##
## locale:
##  [1] LC_CTYPE=en_US.UTF-8	   LC_NUMERIC=C
##  [3] LC_TIME=en_US.UTF-8	   LC_COLLATE=en_US.UTF-8
##  [5] LC_MONETARY=en_US.UTF-8	   LC_MESSAGES=en_US.UTF-8
##  [7] LC_PAPER=en_US.UTF-8	   LC_NAME=C
##  [9] LC_ADDRESS=C		   LC_TELEPHONE=C
## [11] LC_MEASUREMENT=en_US.UTF-8 LC_IDENTIFICATION=C
##
## attached base packages:
##  [1] grid	  parallel   stats4    methods  stats	  graphics  grDevices
##  [8] utils	  datasets   base
##
## other attached packages:
##  [1] Gviz_1.14.0
##  [2] ChIPpeakAnno_3.4.3
##  [3] VennDiagram_1.6.16
##  [4] futile.logger_1.4.1
##  [5] TxDb.Mmusculus.UCSC.mm10.knownGene_3.2.2
##  [6] GenomicFeatures_1.22.7
##  [7] org.Mm.eg.db_3.2.3
##  [8] RSQLite_1.0.0
##  [9] DBI_0.3.1
## [10] AnnotationDbi_1.32.2
## [11] edgeR_3.12.0
## [12] limma_3.26.3
## [13] locfit_1.5-9.1
## [14] statmod_1.4.22
## [15] csaw_1.4.1
## [16] SummarizedExperiment_1.0.1
## [17] Biobase_2.30.0
## [18] rtracklayer_1.30.1
## [19] Rsamtools_1.22.0
## [20] Biostrings_2.38.2
## [21] XVector_0.10.0
## [22] GenomicRanges_1.22.2
## [23] GenomeInfoDb_1.6.1
## [24] IRanges_2.4.6
## [25] S4Vectors_0.8.5
## [26] BiocGenerics_0.16.1
## [27] Rsubread_1.20.2
## [28] knitr_1.11
## [29] BiocStyle_1.8.0
##
## loaded via a namespace (and not attached):
##  [1] httr_1.0.0			regioneR_1.2.0
##  [3] AnnotationHub_2.2.2		splines_3.2.2
##  [5] Formula_1.2-1			shiny_0.12.2
##  [7] interactiveDisplayBase_1.8.0	latticeExtra_0.6-26
##  [9] RBGL_1.46.0			BSgenome_1.38.0
## [11] lattice_0.20-33               	biovizBase_1.18.0
## [13] digest_0.6.8			RColorBrewer_1.1-2
## [15] colorspace_1.2-6               	htmltools_0.2.6
## [17] httpuv_1.3.3			plyr_1.8.3
## [19] XML_3.98-1.3			biomaRt_2.26.1
## [21] zlibbioc_1.16.0              	xtable_1.8-0
## [23] GO.db_3.2.2			scales_0.3.0
## [25] BiocParallel_1.4.3              ggplot2_2.0.0
## [27] nnet_7.3-11			survival_2.38-3
## [29] magrittr_1.5			mime_0.4
## [31] memoise_0.2.1           	evaluate_0.8
## [33] MASS_7.3-45            	   	foreign_0.8-66
## [35] graph_1.48.0			BiocInstaller_1.20.1
## [37] tools_3.2.2			formatR_1.2.1
## [39] matrixStats_0.50.1              stringr_1.0.0
## [41] munsell_0.4.2               	cluster_2.0.3
## [43] ensembldb_1.2.1               	lambda.r_1.1.7
## [45] RCurl_1.95-4.7               	dichromat_2.0-0
## [47] VariantAnnotation_1.16.4        bitops_1.0-6
## [49] gtable_0.1.2			multtest_2.26.0
## [51] R6_2.1.1               		gridExtra_2.0.0
## [53] GenomicAlignments_1.6.1         Hmisc_3.17-1
## [55] futile.options_1.0.0            KernSmooth_2.23-15
## [57] stringi_1.0-1			Rcpp_0.12.2
## [59] rpart_4.1-10			acepack_1.3-3.3
                


For the command-line tools, the fastq-dump utility (version 2.4.2) from the SRA Toolkit must be installed on the system, along with the MarkDuplicates command from the Picard software suite (version 1.117). Readers should note that the read alignment steps for each data set can only be performed on Unix or Mac OS. This is because the various system calls assume that a Unix-style command-line interface is present. In addition, *Rsubread* is not supported for Windows. However, downstream analyses of the BAM files can be performed using any platform on which *R* can be installed. The entire workflow takes 7–8 hours to run and requires 10 GB of RAM.
