# Supplementary material for: From reads to regions: a Bioconductor workflow to detect differential binding in ChIP-seq data
Source: F1000Res. 2016 Jan 11;4:1080. Originally published 2015 Oct 16. [Version 2] doi: 10.12688/f1000research.7016.2 (PMC4706055; doi:10.12688/f1000research.7016.2)
Supplement: Peer review history

# Peer review: Referee response for version 1

**DOI:** 10.5256/f1000research.7553.r10879

**Published:** 2015-12-18

**Authors:** Rory Stark

**Affiliations:** 1 Cancer Research UK Cambridge Institute, University of Cambridge, Cambridge, UK

This article represents a comprehensive and useful presentation of a window-based differential binding analysis. Currently, most quantitative differential binding studies rely on a peak calling step; this demonstration of the benefits of avoiding such a step is of great interest. The workflow is a valuable complement to the author's related published discussions (Lun and Smyth 2014, 2015). It is comprehensive in that is takes the user from archived sequencing reads, through two core analyses, and includes annotation and visualization code as well. Notably the authors include interesting exploration of many important details, particularly in the area of normalization, that are frequently overlooked and can have a crucial impact on analytical results.

I include below a number of questions for the authors, the answer to which may make the article even more useful.

- **Computational resources. **It would be useful to include some discussion of the computational resources required to perform this analysis (memory and compute time). How do memory/compute requirements change as a function of lowering the window size? Can this handle a large number of samples?
- **Experimental design**. Are there any guidelines of how many replicates should be included for a successful analysis? 
- **Duplication rates**. Duplicates are included in the analysis, are there any guidelines for acceptable duplication rates? The authors say that "ideally, the proportion of mapped reads should be high, while the proportion of marked reads should be low" -- how high and how low? Is there some point where there are too many duplicates to expect a successful analysis?
- **Blacklists**. The workflow uses a published blacklist to mask areas of the genome where reads will be not be counted. Some of these are attributable repeat regions, but some are anomalous, and there may be tissue-specific issues. Given that the workflow includes only very limited use of control reads (such as Input) for filtering non-enriched windows, perhaps it would be good to use blacklists generated from the controls, such as those made by the GreyListChIP Bioconductor package.This is especially important for experiments that use multiple tissue types or cell lines.
- **Read counting.** The authors state that “the number of extended reads overlapping a window is counted”. As almost all reads will overlap more than one window (fragment lengths generally being longer than the window size), it would be helpful to be explicit regarding if a read is counted in more than one window. If so, do single reads resulting in multiple counts have an implication for the assumption of binomial distribution of reads?
- **Filtering windows by abundance.**
  - By **using read abundance to remove windows** prior to testing, is there an issue with the same information being used to choosing which windows to compare as is used for the comparison itself? Can window filtering be compared to peak calling in that it uses read counts to reduce the proportion of the genome being considered for differential analysis?
  - By **using a fold measure as a threshold,** in many cases, very small changes in the number of background reads can have a big impact on calculated fold change. How sensitive is this filter process? How important is it to final results?
- **Normalization**
  - Is there way to **determine computationally if a trended-bias normalization is appropriate**, or is this best done by visual examination of the mean-difference plot?
  - If **the scale of differential binding is not known *a priori***, what normalization method should be used? Is it safe to use a non-linear trended correction? Or TMM on large "background" windows?
- **Merging windows**
  - It appears possible to create **merged regions with higher FDR than the minimum FDR of constituent windows**. Windows that would have an FDR lower than some “significance” threshold may be “lost” in a merged region. Is there a way to constrain the merging function to not create a “non-significant” region that contains “significant” windows (according to a specified FDR threshold)?
  - Another reviewer commented that it would be nice to not **merge regions that have windows with both positive and negative fold changes**, this seems useful as well.
  - Regarding regions with positive and negative fold changes, it seems worth referencing MMDiff (Schweikert et al BMC Genomics 2013), which is designed to **detect differences in the gain/loss patterns of binding profiles.**
- **For the CBP example**:
  - One or more **plots visualizing the batch effec**t (MDS/PCA) would be helpful!
  - **Is a batch effect the only possible explanation** for large dispersion estimates and infinite prior d.f.? Can we always assume a batch effect if we see this?
  - If the batch effect applies to a more than one sample, **can this be modeled in a multi-factor design**? If it applies only to one sample, perhaps this is a ChIP efficiency issue?

I have read this submission. I believe that I have an appropriate level of expertise to confirm that it is of an acceptable scientific standard.

## References

[1] SchweikertGCsekeBClouaireTBirdASanguinettiG: MMDiff: quantitative testing for shape changes in ChIP-Seq data sets. *BMC Genomics*.2013;14: 1080 826 10.1186/1471-2164-14-826 24267901PMC4008153

[2] LunATSmythGK: De novo detection of differentially bound regions for ChIP-seq data using peaks and windows: controlling error rates correctly. *Nucleic Acids Res*.2014;42(11) : 1080 e95 10.1093/nar/gku351 24852250PMC4066778

[3] LunATSmythGK: csaw: a Bioconductor package for differential binding analysis of ChIP-seq data using sliding windows. *Nucleic Acids Res*.2015; 1080 10.1093/nar/gkv1191 26578583PMC4797262

# Response

**Published:** 2015-12-19

**Authors:** Aaron Lun

**Affiliations:** Walter and Eliza Hall Institute of Medical Research, Australia

***This article represents a comprehensive and useful presentation of a window-based differential binding analysis.***

Thanks Rory.

***It would be useful to include some discussion of the computational resources required to perform this analysis (memory and compute time). How do memory/compute requirements change as a function of lowering the window size? Can this handle a large number of samples?***

Including the alignment steps, the entire analysis of the two data sets takes approximately 7-8 hours. It uses around 10 GB of memory in total, most of which is spent in alignment or in processing the large numbers of windows in edgeR. Memory requirements will not change as a function of window size but will change linearly as a function of window spacing, as this determines the number of windows that are extracted from the genome. We recommend that, for large windows, the spacing can be increased to avoid unnecessary computational work (given that any additional loss of spatial resolution is irrelevant for large windows). As for the number of samples, we typically use csaw to analyze data from small contained experiments (5-10 samples). Large numbers (>50) of samples will probably require high-performance computing resources and some additional care, e.g., chromosome-by-chromosome processing. As suggested, we have added some expected time/memory requirements to the "Software availability" section.

***Are there any guidelines of how many replicates should be included for a successful analysis?***

edgeR requires at least two replicates in one group to estimate the NB/QL dispersion. We usually analyze data with two replicates in each of our biological conditions of interest. This ensures that the results for each condition are replicable and are not the result of technical artifacts such as PCR duplicates. We have added some comments regarding this to the text. Of course, the more replicates, the better, but we appreciate that ChIP-seq is one of the more technically challenging techniques and that obtaining high levels of replication is not always feasible.

***Duplicates are included in the analysis, are there any guidelines for acceptable duplication rates? The authors say that "ideally, the proportion of mapped reads should be high, while the proportion of marked reads should be low" -- how high and how low? Is there some point where there are too many duplicates to expect a successful analysis?***

We have added some ballpark figures to the workflow. In our experience, mapping proportions above 70 to 80% are quite satisfactory (with the missing reads probably lying in unmappable repeat regions), along with duplicate proportions below 20%. While some of the marked reads will inevitably correspond to non-duplicate fragments that happen to overlap in enriched regions, this is unlikely to explain very high proportions of marked reads (> 40%). Such cases are more likely to be caused by high levels of PCR duplication.

***Given that the workflow includes only very limited use of control reads (such as Input) for filtering non-enriched windows, perhaps it would be good to use blacklists generated from the controls, such as those made by the GreyListChIP Bioconductor package.This is especially important for experiments that use multiple tissue types or cell lines.***

Done.

***As almost all reads will overlap more than one window (fragment lengths generally being longer than the window size), it would be helpful to be explicit regarding if a read is counted in more than one window. If so, do single reads resulting in multiple counts have an implication for the assumption of binomial distribution of reads?***

Yes, reads are often counted in more than one window. We have added a statement regarding this to the workflow. Multiple counting of reads introduces technical correlations between windows, but this will not affect the downstream analysis. Estimation of the EB shrinkage statistics in edgeR are robust to correlations between features. Similarly, the BH correction used to control the FDR is robust to correlations between tests.

***By using read abundance to remove windows prior to testing, is there an issue with the same information being used to choosing which windows to compare as is used for the comparison itself? Can window filtering be compared to peak calling in that it uses read counts to reduce the proportion of the genome being considered for differential analysis?***

The use of an independent filtering criterion is the important concept here. In a NB model, the average abundance (i.e., the log-NB mean) is a filter statistic that is independent of the DB status of each test, i.e., knowing the average abundance doesn't tell you whether or not the window is DB. This ensures that the selection of high-abundance windows will not bias the analysis towards or against detecting DB in those windows, and maintains the validity of the downstream multiplicity correction procedures.

Conceptually, filtering and peak calling have some similarities, as you have pointed out. However, from a statistical perspective, filtering on the average abundance is only equivalent to peak calling if all libraries were pooled together and the pooled library was used for peak calling (and even then, only if those libraries are of the same size). Indeed, most peak callers were not designed for processing multi-sample data sets, especially not in a manner that preserves the validity of downstream DB analyses.

***By using a fold measure as a threshold, in many cases, very small changes in the number of background reads can have a big impact on calculated fold change. How sensitive is this filter process? How important is it to final results?***

We protect against this effect by using large bins to compute the background abundance. This increases the number of reads in each bin and stabilizes the estimated abundance against stochastic changes in coverage. Further stability is provided by taking the median of the abundance across all bins. We find that random differences in coverage make little difference to the estimated background and to the filter threshold. Instead, filtering is more sensitive to the choice of the fold-increase over the background. Obviously, requiring a larger fold-increase will result in the loss of weak binding sites. This is an arbitrary decision that depends on what the user considers to be relevant and cannot be avoided, even with peak callers (in which case, the choice of threshold is that of the significance of each peak).

***Is there way to determine computationally if a trended-bias normalization is appropriate, or is this best done by visual examination of the mean-difference plot?***

This is best done with visual examination. Abundance-dependent normalization involves fitting a trend, and then adjusting the observations in order to "flatten out" the trend. This means that if you were to repeat the trend fitting process on the adjusted data, you would end up with a flat trend by definition. This would not be helpful in diagnosing problems in the normalization procedure.

***If the scale of differential binding is not known a priori, what normalization method should be used? Is it safe to use a non-linear trended correction? Or TMM on large "background" windows?***

We would suggest repeating the analysis with both normalization strategies. If they give similar results, then it doesn't matter which method is used. However, if they yield different results, this indicates that there are systematic differences in read coverage between some of the libraries. The origin of these differences cannot be conclusively determined from the data alone - they may be due to differences in IP efficiency, or due to systematic and genuine DB in one set of conditions. Thus, care will be required in the interpretation of the DB results. Regions detected with both approaches are most likely to be reliable.

We note that systematic differences between replicates within a condition can be directly interpreted as efficiency biases. However, this does not guarantee that there are no systematic differences in binding between conditions. Applying normalization to remove efficiency biases will also remove any systematic and genuine DB between conditions. In short, an assumption about the underlying biology is still required in order to apply one method or the other. In practice, normalization of the efficiency biases is usually the lesser of two evils, as large differences between replicates will inflate the dispersions and interfere with DB detection.

A third possibility is to use spike-ins (e.g., *Drosophila* chromatin) for normalization. This should be able to distinguish between genuine DB and efficiency bias, as only the latter should affect spike-in coverage. See the csaw user's guide for some guidelines on accommodating spike-in data in csaw.

***Is there a way to constrain the merging function to not create a “non-significant” region that contains “significant” windows (according to a specified FDR threshold)?***

As a general rule, the merging algorithm must be independent of the significance of the window in order to maintain statistical validity. This means that it is not allowed to know which windows are significant or not during its operation. If this is not true, FDR control across the regions cannot be guaranteed.

That said, it may be useful in some applications to get tighter intervals containing only the significantly DB regions. This can be achieved by defining putative DB windows based on a window-level FDR threshold, and then clustering them to obtain DB regions. An informal estimate of the region-level FDR for these DB regions can be computed using the clusterFDR() function, and the window-level threshold can then be adjusted until the region-level FDR is below some desired threshold. This two-step procedure is necessary as the window-level and region-level FDRs can be quite different for many overlapping windows. However, it is less statistically rigorous than the default approach which is blind to the significance of each window.

***Another reviewer commented that it would be nice to not merge regions that have windows with both positive and negative fold changes, this seems useful as well.***

Such a merging procedure would result in loss of detection power - see our comments below.

***One or more plots visualizing the batch effect (MDS/PCA) would be helpful!***

Added. Note that we use a larger 'top' set of windows to make the MDS plot. This is simply to improve the visualization of the systematic differences between libraries that are not captured with the default top value (500).

***Is a batch effect the only possible explanation for large dispersion estimates and infinite prior d.f.? Can we always assume a batch effect if we see this?***

Very large dispersions in conjunction with infinite prior d.f. means there are large differences between the replicates that are too consistent to be random. In other words, there are systematic differences between the replicates, and that is almost the definition of a batch effect.

***If the batch effect applies to a more than one sample, can this be modeled in a multi-factor design? If it applies only to one sample, perhaps this is a ChIP efficiency issue?***

Yes, the batch effect can easily be modelled in the GLM, provided that it does not confound detection of DB between the conditions of interest.

Batch effects and differences in ChIP efficiency are not mutually exclusive - the fact that samples are processed in separate batches is often the cause for a difference in efficiency. In this case, systematic differences in peak heights are observed between the two CBP WT replicates. This can be seen most clearly in the coverage tracks of Figure 12 (13 in version 2). This would suggest that the batch effect corresponds to an increase in ChIP efficiency for one of the WT libraries.

# Peer review: Referee response for version 1

**DOI:** 10.5256/f1000research.7553.r10877

**Published:** 2015-11-25

**Authors:** Cenny Taslim

**Affiliations:** 1 Comprehensive Cancer Center, Ohio State University Medical Center, Columbus, OH, USA

This article provides a useful workflow on detecting differential binding in ChIP-seq data with examples and codes from R packages and other softwares. This paper will help researchers on analyzing their ChIP-seq using R. However, it would be better if there was more description on the methods used in the workflow.

“Reads are first aligned to the genome using the *Rsubread* package ( Liao *et al.*, 2013).” Please describe what algorithm is used to align the reads.

"Technical replicates are merged together prior to further processing." What does merged together mean? Concatenate? Or average? Or pick one randomly?

"Ideally, the proportion of mapped reads should be high, while the proportion of marked reads should be low." Proportion mapped is those reads that can be uniquely mapped to the genome?

"Thus, the marking status of each read will be ignored in the rest of the analysis, i.e., no duplicates will be removed in downstream steps." I believe generally people exclude duplicates in downstream steps?

"By default, windows with very low counts are removed to reduce memory use." What is the definition of very low counts? <=1? If it is described by the section filtering windows by abundance, please mention it.

bins <- windowCounts(bam.files, bin=TRUE, width=2000, param=param)

filter.stat <- filterWindows(win.data,  bins, type="global")

min.fc <- 3

keep <- filter.stat$filter > log2(min.fc)

summary(keep)

##     Mode      FALSE    TRUE   NA's

##  logical   906406 663218     0

hist(filter.stat$back.abundances, xlab="Background abundance", main="", breaks=50)

threshold <- filter.stat$abundances[1] - filter.stat$filter[1] + log2(min.fc)

abline(v=threshold, col="red")

In code above, where is the background? I assumed it is filter.stat$filter[1]? However, it looks like the filter is background + log2(3)? If it’s at least 3-fold background, shouldn’t it be filter.stat$filter[1]*3?

Figure 2 doesn’t make sense. Why is there windows with < 0 abundance?

Normalizing for library-specific trended biases. Figure 3 only shows log-fold change between mature B and pro-B but there is more than one sample in mature B and pro-B. How do you apply the normalization? Do you take average of all mature B samples and average of all pro-B samples and then do the loess normalization?

##     Gene nWindows logFC.up logFC.down    PValue        FDR

## 6  Ldlrap1     19      11              0 0.224741404877 0.2705479855

## 7  Mdn1        29      12              11 0.000004447727 0.0001347924

## 8  Pydc3        8        0             6 0.051183399851 0.0781822366

## 9  Wfdc17       6       0              6 0.000069604922 0.0008738790

## 10 Mfap1b      19       1              10 0.107116609335 0.1440819313

## 13 Gm15772     30      12              7 0.085543435687 0.1192823092

How do you interpret this results? For Mdn1, there are 29 DB windows? What are the logFC.up and logFC.down.

I have read this submission. I believe that I have an appropriate level of expertise to confirm that it is of an acceptable scientific standard, however I have significant reservations, as outlined above.

# Response

**Published:** 2015-12-03

**Authors:** Aaron Lun

**Affiliations:** Walter and Eliza Hall Institute of Medical Research, Australia

Thank you for your report. You asked for more details of the methodology in a number of places, and we have added further details as appropriate. We are puzzled about your approval "with reservations", as we did not find any criticisms in your report, apart from the Figure 2 x-label not being explicit about the log-scale used.

***“Reads are first aligned to the genome using the Rsubread package (Liao et al., 2013).” Please describe what algorithm is used to align the reads.***

The alignment algorithm in Rsubread package uses a seed-and-vote paradigm. We have added a very brief mention of this, though for a complete description of the algorithm, it would be advisable to consult the Liao et al. reference. We note that the differences between different aligners are not relevant to this workflow article. What is relevant is that the subread aligner is an appropriate aligner for ChIP-seq data, and that it is available as a native implementation in a Bioconductor package.

***"Technical replicates are merged together prior to further processing." What does merged together mean? Concatenate? Or average? Or pick one randomly?***

Merging simply refers to pooling the reads from all replicates into a single library. We have altered the wording to make this more obvious.

***"Ideally, the proportion of mapped reads should be high, while the proportion of marked reads should be low." Proportion mapped is those reads that can be uniquely mapped to the genome?***

Yes. By default, Rsubread only reports mapped reads as those with unique mapping locations. We have added a mention of this to the text.

***"Thus, the marking status of each read will be ignored in the rest of the analysis, i.e., no duplicates will be removed in downstream steps." I believe generally people exclude duplicates in downstream steps?***

Duplicate removal is not recommended for routine DB analyses with edgeR. It will reduce detection power by capping read coverage at strongly bound sites, such that a region that is DB may not be detected if the coverage of that region gets capped to the same level in two libraries. It may also increase false positives for analyses involving different library sizes, when the same cap is applied across libraries; subsequent normalization for library size will result in a spurious difference in the capped heights.

In theory, duplicate removal should only be necessary and appropriate when a large portion of duplicate reads arise from PCR duplicates of the same DNA fragment. This should seldom occur unless the amount of DNA is overly small, in which case there are likely to be more general problems with data quality.

***"By default, windows with very low counts are removed to reduce memory use." What is the definition of very low counts? <=1? If it is described by the section filtering windows by abundance, please mention it.***

The internal filter in windowCounts removes windows with count sums less than 10 across all libraries, simply to reduce memory usage. Windows with such low counts will not provide sufficent evidence for detecting DB, so their removal does not seem like a major loss. We have added a mention of this threshold to the text.

In code above, where is the background? I assumed it is filter.stat$filter[1]? However, it looks like the filter is background + log2(3)? If it’s at least 3-fold background, shouldn’t it be filter.stat$filter[1]*3?

By default, abundances in edgeR are reported as log-CPMs. So, a 3-fold increase over the background coverage corresponds to a log2(3) increase in the abundance. This has been reworded for more clarity.

***Figure 2 doesn’t make sense. Why is there windows with < 0 abundance?***

Again, abundances are reported as log-CPMs, for which it is entirely possible to obtain negative values. We have clarified this on the x-axis label.

***Normalizing for library-specific trended biases. Figure 3 only shows log-fold change between mature B and pro-B but there is more than one sample in mature B and pro-B. How do you apply the normalization? Do you take average of all mature B samples and average of all pro-B samples and then do the loess normalization?***

The algorithm constructs an average library containing average counts across all samples for each window. It then performs loess normalization between each sample and this average sample. A full description is available in our recently published paper (doi: 10.1093/nar/gkv1191, to which we have added a reference), but is beyond the scope of this workflow article.

***How do you interpret this results? For Mdn1, there are 29 DB windows? What are the logFC.up and logFC.down.***

We have already described the meaning of these fields in the text following the first call to combineTests(), in the section "Controlling the FDR across regions". The same interpretation can be applied to the output of all combineTests() calls.

# Peer review: Referee response for version 1

**DOI:** 10.5256/f1000research.7553.r10876

**Published:** 2015-10-30

**Authors:** Lihua Julie Zhu, Jianhong Ou

**Affiliations:** 1 Department of Molecular, Cell and Cancer Biology, Program in Bioinformatics and Integrated Biology, Program in Molecular Medicine, University of Massachusetts Medical School, Worcester, MA, USA; 2 Department of Molecular, Cell and Cancer Biology, University of Massachusetts Medical School, Worcester, MA, USA

This article “From reads to regions: a Bioconductor workflow to detect differential binding in ChIP-seq data” clearly describes a comprehensive computational workflow of Differential Binding (DB) analysis of ChIP-seq data set, based primarily on R software packages from the open-source Bioconductor project. It provides readers with practical usage examples of DB analyses of two typical types of ChIP-seq data sets, that covers all steps of the analysis pipeline, from alignment of read sequences, normalization, DB identification, annotation to interpretation and visualization of putative DB regions. We believe that this well-written paper will greatly help users to apply the workflow to their own DB analysis of ChIP-seq data sets.

In the following section, please explicitly explain that the two data sets were chosen to represent two common ChIP-seq use cases, one data set for dealing with wider peaks while the other data set is for working with sharp peaks

"The application of the methods in this article will be demonstrated on two publicly available ChIP-seq data sets. The first data set studies changes in H3K9ac marking between pro-B and mature B cells (Revilla-I-Domingo *et al.*, 2012). The second data set studies changes in CREB-binding protein (CBP) binding between wild-type and CBP knock-out cells (Kasper *et al.*, 2014). "

According to the Rsubread documentation about parameter ***type ***at

http://bioconductor.org/packages/release/bioc/manuals/Rsubread/man/Rsubread.pdf, ***type ***is an integer giving the type of sequencing data. Possible values include ***0 (RNA- seq data) and 1 (genomic DNA-seq data such as WGS, WES, ChIP-seq data etc.).  By default, it is set to 0.  ***Therefore, please set type to 1 in the following section.

"

library(Rsubread)

bam.files <- paste0(names(by.group), ".bam")

align(index=“index/mm10", readfile1=group.fastq, TH1=2,

    input_format=“FASTQ", output_file=bam.files)

"

"align(index="index/mm10", readfile1=all.fastq, phredOffset=64,

     input_format="FASTQ", output_file=bam.files)"

Suggest adding file.exists check to see if temp.dir exists already before dir.create(temp.dir).

For system call to fastq-dump and MarkDuplicates, suggest to add path information to these programs in case the path to these programs are not in the search path.

"For this analysis, reads are only used if they have a mapping quality score above 50." 

50 is pretty high although we understand that this is for illustration purposes. To prevent readers from getting the idea that 50 is the recommended cutoff, could you please also provide a quality score threshold commonly used in the field?

"The filter threshold is defined based on the assumption that most regions in the genome are not marked by H3K9ac. Reads are counted into large bins and the median coverage across those bins is used as an estimate of the background abundance. Windows are only retained if they have abundances 3-fold higher than the background."  

In the example, the window size (width) for calculating background (bg) coverage is set to 2000 bp, and the width is set to 150 bp for win.data. When calculating fold enrichment over background, is the bg coverage scaled down using the ratio of the window size (2000 vs. 150)?

"The implicit assumption of non-linear methods is that most windows at each abundance are not DB. Any systematic difference between libraries is attributed to bias and is removed. This is not appropriate in situations where large-scale DB is expected, as removal of the difference would result in loss of genuine DB. However, there is no indication that such changes are present in this data set, so non-linear methods can be applied without too much concern."

Could you please suggest a normalization method for situations where large-scale DB is expected (perhaps TMM as mentioned in the later section)?

"Note that only the trended dispersion will be used here – the common and tagwise values are only shown for diagnostic purposes"

Could you please comment on how common and tag wise values help with diagnosis of the data set?

"Determining the direction of DB is more complicated, as clusters could potentially contain windows that are changing in opposite directions."

How about controlling this by allowing nearby windows to merge only if the changing directions are the same?

"The behaviour of *ChIPpeakAnno* complements that of detailRanges. The latter reports all overlapping and flanking genes, while the former reports only the closest gene (but in greater detail). Which is preferable depends on the proclivities of the user and the purpose of the annotation."

Actually, ChIPpeakAnno can also report all overlapping and flanking genes by setting output="both" (or output ="overlapping") and maxgap. For example, it outputs all overlapping and flanking genes within 5kb if set maxgap = 5000L and output ="overlapping".  Here is an example using Txdb annotation data.

library(TxDb.Mmusculus.UCSC.mm10.knownGene)

ucsc.mm10.knownGene <- genes(TxDb.Mmusculus.UCSC.mm10.knownGene)

anno.peaks <- annotatePeakInBatch(minimal, annotationData=ucsc.mm10.knownGene, 

              output="overlapping", maxgap=5000L)

"Reads are then counted into sliding windows. For TF data analyses, smaller windows are necessary to capture sharp binding sites. A large window size will be suboptimal as the count for a particular site will be “contaminated” by non-specific background in the neighbouring regions. In this case, a window size of 10 bp is used.

win.data <- windowCounts(bam.files, param=param, width=10, ext=frag.len)"

It seems that space is set to 50bp for both sharp peaks and broad peaks. Could you please comment on this? 

We notice that the width for local background is set to 2kb for broad peaks and 10kb for sharp peaks. Could you please justify? Would adding a fitted line in Figure 3 and Figure 4 help with the visualization? In addition, it would be great if you could provide recommendations on how to evaluate the quality of the BCV in Figure 5. Is there a range or specific shape we are targeting?

We have read this submission. We believe that we have an appropriate level of expertise to confirm that it is of an acceptable scientific standard.

# Response

**Published:** 2015-11-02

**Authors:** Aaron Lun

**Affiliations:** Walter and Eliza Hall Institute of Medical Research, Australia

***We believe that this well-written paper will greatly help users to apply the  workflow to their own DB analysis of ChIP-seq data sets.***

Thanks Julie.

***In the following section, please explicitly explain that the two data sets  were chosen to represent two common ChIP-seq use cases, one data set for dealing with wider peaks while the other data set is for working with sharp peaks***

Done.

***Therefore, please set type to 1 in the following section.***

Done. We note that, at the time of publication, the workflow was constructed using packages in BioC 3.1, at which time the "type" parameter was not available in Rsubread. This has now been changed, along with minor code updates to various other parts of the workflow for BioC 3.2.

***Suggest adding file.exists check to see if temp.dir exists already before  dir.create(temp.dir).***

There's no need for the extra check. By default, "dir.create" gives a warning if the directory already exists. We want users to be able to run the code multiple times if they wish, overwriting earlier files if they exist.

***For system call to fastq-dump and MarkDuplicates, suggest to add path information to these programs in case the path to these programs are not in the search path.***

We believe that implementing this suggestion may be counter-productive for most readers; the absolute path to these programs on our machines will not be useful to the general audience, given that the installation directory will change on different machines. It is the user's responsibility to make sure that these system commands are available prior to analysis - how this is done is largely irrelevant to successful execution of the workflow. The Picard software suite and the SRA Toolkit are quite widely used, so we do not think that installation and access would pose a major problem for most users.

***To prevent readers from getting the idea that 50 is the recommended cutoff,  could you please also provide a quality score threshold commonly used in the field?***

We use 50 for the first data set as the reads are very short (< 40 bp) such that strict filtering is required to avoid mapping errors, non-uniquely-mapped reads, etc. We use the same value for the second data set for consistency, though we concede that a lower value could be used in practice. We have added a comment regarding this to the workflow.

We also note that the MAPQ values reported by (R)subread tend to be quite binary for long reads, i.e., either very low or very high. For example, in SRR1145790, there are 24212282 reads with a MAPQ score >= 50, and 24907156 with a MAPQ score >= 10. This equates to a (relatively minor) 3% increase in available reads from a large change in the MAPQ threshold. All in all, we believe that analyses based on Rsubread alignments are generally robust to the choice of threshold.

***When calculating fold enrichment over background, is the bg coverage scaled  down using the ratio of the window size (2000 vs. 150)?***

Yes. This is done automatically within the "filterWindows" function. We have added a note to the workflow to point this out.

***Could you please suggest a normalization method for situations where  large-scale DB is expected (perhaps TMM as mentioned in the later section)?***

As you have noted, this issue is addressed more comprehensively in the analysis for the CBP data set. We have added a comment here regarding the existence of alternative normalization strategies when large-scale DB is expected.

***Could you please comment on how common and tag wise values help with diagnosis of the data set?***

The common BCV provides a measure of the overall variability of the data set, averaged across all windows. The tagwise BCVs should be dispersed around the fitted trend to indicate that the fit was successful. These comments have been added to the workflow.

***How about controlling this by allowing nearby windows to merge only if the changing directions are the same?***

This possibility has not escaped us. However, the sign of the log-FC is not independent of the DB status of each window in this particular application. In fact, clustering windows based on the signs of the log-FCs will result in loss of detection power for DB events.

To illustrate, consider a non-DB site with a number of overlapping windows. Due to stochasticity, the log-FCs of those windows fluctuate around zero. Now, assume that we only allow merging of adjacent windows where the signs of the log-FCs are identical. For our non-DB site, we end up with lots of small clusters, comprised of windows with positive or negative log-FCs due to chance. In contrast, for a genuinely DB site, all windows will have positive (or negative) log-FCs, as stochasticity will not change the sign of the log-FC.

When this behaviour is considered on a genome-wide level, we can see that non-DB sites will generate several non-DB clusters, while DB sites will only generate one DB cluster. This results in an increase in the number of tests with large p-values, increasing the severity of the BH correction without any increase in the number of potential discoveries. Ultimately, this results in the loss of detection power for DB events. One might be tempted to overcome this by filtering out small non-DB clusters, but this will compromise FDR control and result in liberalness.

We consider loss of power to be more problematic than the existence of complex DB events. The former means that you don't detect anything, whereas the latter just requires some more consideration during interpretation. In fact, proper identification of complex events is not irrelevant - the context of a change in binding will affect its interpretation (e.g., if it occurs adjacent to an opposing change in the same general region) and this would be lost if changes were reported separately.

Of course, these theoretical issues are beyond the scope of this workflow article. However, considerations of proper FDR control across regions have been discussed in our methodological articles (Lun and Smyth, NAR 2014; Lun and Smyth, NAR 2015).

***Actually, ChIPpeakAnno can also report all overlapping and flanking genes by setting output="both" (or output ="overlapping") and maxgap. For example, it outputs all overlapping and flanking genes within 5kb if set maxgap = 5000L and output ="overlapping".***

Thanks for letting us know. We have amended our comment regarding the differences between the two annotation strategies. We note that there are still some differences; in particular, for any given input region, "annotatePeakInBatch" with output="overlapping" reports each overlapping feature as a separate entry of the output object, while "detailRanges" reports all overlapping features in one string. The former provides more detail and is easier to use for further analysis, while the latter is more convenient for annotating an existing DB list that needs to be saved to file.

***It seems that space is set to 50bp for both sharp peaks and broad peaks. Could you please comment on this?***

In general, the spacing governs the compromise between spatial resolution and computational convenience. Smaller spacings increase resolution, at the cost of increasing memory usage and runtime. However, for most ChIP-seq analyses, resolution is fundamentally limited by the width of the binding event and by the size of the post-sonication fragments. As long as the spacing is smaller than either of these two parameters (i.e., the window size and the extension length), there will be enough windows to profile each interval of the genome for a satisfactory analysis. Smaller spacings and additional windows will provide no benefit, and will only increase computational work. This is true for both TF and histone mark analyses, such that the choice of spacing interval does not need to be changed between them. We have added a brief remark about this to the workflow.

***We notice that the width for local background is set to 2kb for broad peaks and 10kb for sharp peaks. Could you please justify?***

A local background of 2 kbp can also be used for sharp peaks. We have used 10 kbp for convenience; the counts for large bins were already loaded for normalization in the previous step, so it makes sense to re-use them for filtering. In general, smaller bins provide a more accurate estimate of the background abundance, as unbound regions can be distinguished from binding sites more effectively. However, loss of spatial resolution is negligible for large background regions in the CBP data set. We have added a comment about this to the workflow.

***Would adding a fitted line in Figure 3 and Figure 4 help with the visualization?***

We believe that the trend in the log-fold changes is obvious enough without the need for a fitted line.

***In addition, it would be great if you could provide recommendations on how to evaluate the quality of the BCV in Figure 5. Is there a range or specific shape we are targeting?***

As a general rule, we expect to see a curve that decreases to a plateau with increasing average abundance. This reflects the increased reliability of the data at large counts, where the effects of stochasticity and technical artifacts (e.g., mapping errors, PCR duplicates) are averaged out. In Figure 5, the range of abundances is such that the plateau has already been reached; a more dramatic decrease can be observed by including more lower-abundance windows.
